# RosettaHDX: Predicting antibody-antigen interaction from hydrogen-deuterium exchange mass spectrometry data

**DOI:** 10.1016/j.jsb.2025.108166

**Published:** 2025-01-05

**Authors:** Minh H. Tran, Cristina E. Martina, Rocco Moretti, Marcus Nagel, Kevin L. Schey, Jens Meiler

**Affiliations:** a Chemical and Physical Biology Program, Vanderbilt University, Nashville, TN, USA; b Center of Structural Biology, Vanderbilt University, Nashville, TN, USA; c Department of Chemistry, Vanderbilt University, Nashville, TN, USA; d Mass Spectrometry Research Center, Department of Biochemistry, Vanderbilt University, Nashville, TN, USA; e Institute for Drug Discovery, Institute for Computer Science, Wilhelm Ostwald Institute for Physical and Theoretical Chemistry, University Leipzig, Leipzig, Germany; f Center for Scalable Data Analytics and Artificial Intelligence ScaDS.AI and School of Embedded Composite Artificial Intelligence SECAI, Dresden/Leipzig, Germany; g Department of Pharmacology, Institute of Chemical Biology, Center for Applied Artificial Intelligence in Protein Dynamics, Vanderbilt University, Nashville, TN, USA

**Keywords:** Hydrogen–deuterium exchange mass spectrometry (HDX-MS), Antibody-antigen interaction, Epitope-paratope identification, Protein–protein docking, Structure modeling, Integrative structural biology

## Abstract

High-throughput characterization of antibody-antigen complexes at the atomic level is critical for understanding antibody function and enabling therapeutic development. Hydrogen-deuterium exchange mass spectrometry (HDX-MS) enables rapid epitope mapping, but its data are too sparse for independent structure determination. In this study, we introduce RosettaHDX, a hybrid method that combines computational docking with differential HDX-MS data to enhance the accuracy of antibody-antigen complex models beyond what either method can achieve individually. By incorporating HDX data as both distance restraints and a scoring term in the Rosetta-Dock algorithm, RosettaHDX successfully generated near-native models (interface root-mean square deviation ≤ 4 Å) for all 9 benchmark complexes examined, averaging 3.6 times more near-native models than Rosetta alone. Near-native models among the top 10 scoring were identified in 3/9 cases, compared to 1/9 with Rosetta alone. Additionally, we developed a predictive metric based on docking results with HDX restraints to identify allosteric peptides in HDX datasets.

## 1. Introduction

Antibodies (Abs) are crucial components of the adaptive immune system, defending the host from threats such as viruses. Utilizing hypervariable complementarity-determining regions (CDRs), Abs can recognize and bind to a vast array of viral proteins with high specificity (Sela-Culang et al., 2013b). Given the highly specific and diverse nature of antibody–antigen (Ab-Ag) interactions, high-resolution structures of Ab-Ag complexes are critical for understanding viral immune responses (Williamson et al., 2021) and developing structure-based antiviral therapeutics (Engdahl et al., 2023; Kwong et al., 2020; Sevy et al., 2019). More specifically, insights into the molecular mechanisms of Ab recognition of viral epitopes, derived from these structures, can guide rational vaccine design by focusing efforts on key epitopes (Burton, 2017; Correia et al., 2014; Martina et al., 2023; Rappuoli et al., 2016). This knowledge also facilitates more efficient optimization of Abs for improved breadth and affinity (Hummer et al., 2022; Ward and Wilson, 2020).

To advance next-generation Ab and vaccine development, there is a growing demand for high-throughput generation of precise Ab-Ag complex structures, exceeding the capabilities of traditional methods (Graham et al., 2019; Hederman and Ackerman, 2023). Commonly-used high-resolution techniques such as cryo-electron microscopy or X-ray crystallography are often time- and resource-intensive, have variable success rates, and miss certain dynamic aspects of the interaction (Ziegler et al., 2021). In the field of high-throughput epitope mapping studies, hydrogen–deuterium exchange mass spectrometry (HDX-MS) has emerged as a valuable method with increasing widespread use (Masson et al., 2019; Ständer et al., 2021). With recent advances in methodology and instrumentation (James et al., 2021), HDX-MS offers several advantages that are ideal for epitope mapping including: quick sample preparation, minimal sample requirements (microliters at low micromolar concentrations), fast turnaround time (as little as a week), and virtually no protein size limitations (Jethva and Gross, 2023; Pandit et al., 2012).

By comparing the deuterium uptake of digested Ag peptides between their bound and unbound forms over a labeling time course, HDX-MS provides insights into potential peptides involved in Ab-Ag interactions. Upon complexation, peptides at direct binding sites typically show significant decreases in deuterium uptake. However, complexation can also induce conformational changes away from the binding sites, leading to decreased deuterium uptake in peptides at non-binding sites (referred to as allosteric peptides). Distinguishing these allosteric peptides (which represent false-positive binding site signals) from true interacting peptides can be challenging without additional complementary experiments, highlighting an inherent limitation of HDX-MS (Tran et al., 2022). Computational methods can offer orthogonal and confirmatory support to elucidate which peptides are part of the binding sites and which are allosteric. Additionally, while HDX-MS data can be sparse, integrating it with computational methods presents a promising approach for systematically elucidating Ab-Ag complexes at the atomic level.

Modern computational methods have achieved substantial success in predicting protein complexes. The release of AlphaFold (AF) (Jumper et al., 2021) and AF-Multimer (Evans et al., 2022), deep neural network-based models that leverage sequence coevolution data, along with multiple advance deep learning methods, have enabled unprecedented accuracy in *de novo* protein structure prediction (Baek et al., 2021). Although AF-Multimer’s performance on Ab-Ag complexes remains suboptimal due to the lack of effective coevolutionary signals, its near-native modeling success rate has progressively improved since its initial release (Yin et al., 2022). The success rate has increased to over 30 % in the latest open-source version (v.2.3) and to approximately 60 % in the most recent AF3 release (Abramson et al., 2024; Yin and Pierce, 2024). However, implementing AF3 is challenging because large-scale sampling, an important factor for its success as emphasized by the authors, is currently limited by server constraints, which allow only a maximum of 100 output models per day (Abramson et al., 2024).

Traditional protein–protein docking offers an alternative computational approach for predicting complex models. Typically, protein docking methods use the structures of individual binding partners as input, sample a wide range of their favorable relative orientations, and evaluate these conformations with a scoring function. The accuracy of docking scoring and sampling greatly benefits from incorporating sparse experimental data. Among various successful docking methods (e.g., ClusPro (Comeau et al., 2004; Kozakov et al., 2017), HDOCK (Yan et al., 2020; Yan et al., 2017), ZDOCK (Pierce et al., 2011), SwarmDock (Torchala et al., 2013), HADDOCK (de Vries et al., 2010; Dominguez et al., 2003b; van Zundert et al., 2016), RosettaDock, part of the Rosetta molecular modeling suite, has been extensively coupled with experimental data and proven effective for this purpose (Chaudhury et al., 2011; Lyskov and Gray, 2008; Marze et al., 2018). Over the years, it has achieved successes in predicting protein complex structures using sparse data from numerous experimental techniques such as chemical cross-linking, EPR/DEER, SID-MS, SAXS, FRET, cryo-EM, and covalent labeling methods (Seffernick and Lindert, 2020). However, a hybrid method that incorporates HDX-MS data into the RosettaDock framework to facilitate protein complex prediction, specifically for Ab-Ag complexes, has not yet been developed.

To address this gap, we developed a methodology named RosettaHDX that integrates HDX-MS data into the RosettaDock protocol, offering a robust approach for reliable, high-throughput Ab-Ag complex characterization. Although HDX-MS data have been applied as spatial restraints to guide docking algorithms in several epitope mapping studies (Bennett et al., 2019; Deng et al., 2017; Fields et al., 2021; Huang et al., 2018; Pandit et al., 2012), there has not been an established best practice to define these restraints (Tran et al., 2022). This is crucial because the geometric distances inferred from conventional HDX-MS experiments are often ambiguous, unlike the fixed-length distance measurements between specific cross-linked residues provided by chemical cross-linking. In addition, there has not yet been a benchmark study to establish an optimal protocol for combining HDX-MS and computational docking and evaluating their performance in predicting Ab-Ag complexes. In this study, we propose a refined setup for HDX-guided distance restraints specifically tailored for Ab-Ag docking. Penalty scores derived from the restraints are used to guide model generation and are combined with the Rosetta interface score to guide model selection. Our benchmark set consists of nine Ab-Ag complexes targeting influenza hemagglutinin, with known structures and HDX-MS data. The tolerance of RosettaHDX towards false positive data points (i.e., allosteric peptides) is explored. Next, we evaluate the performance of RosettaHDX in a more realistic scenario by using AF models of unbound Abs and Ags as docking inputs, and compare it to the state-of-the-art AF-Multimer method. Lastly, we develop a predictive metric to identify allosteric peptides in the HDX dataset based on the docking results with HDX restraints.

## 2. Results and discussion

### 2.1. Generating baseline docking performance using RosettaDock

To establish RosettaDock’s baseline global docking performance without HDX-MS data, we generated sets of 10,000 models for each of the nine benchmark complexes. These complexes include Abs targeting various strains and subtypes of influenza hemagglutinin: H7.167, H7–200, and H7.5 binding to H7; H5.28, H5.3, and H5.31 binding to H5; FluA-20 binding to H1; FI6V3 binding to H3 (referred to here as FI6V3_3ztj); and FI6V3 binding to H1 (referred to here as FI6V3_3ztn). All complexes have experimentally determined structures and HDX-MS epitope mapping data available. Details of the benchmark dataset and corresponding HDX results are in Table S1 and Fig. S1.

### 2.2. Converting HDX-MS data to restraints in RosettaHDX

**Fig. 1.**
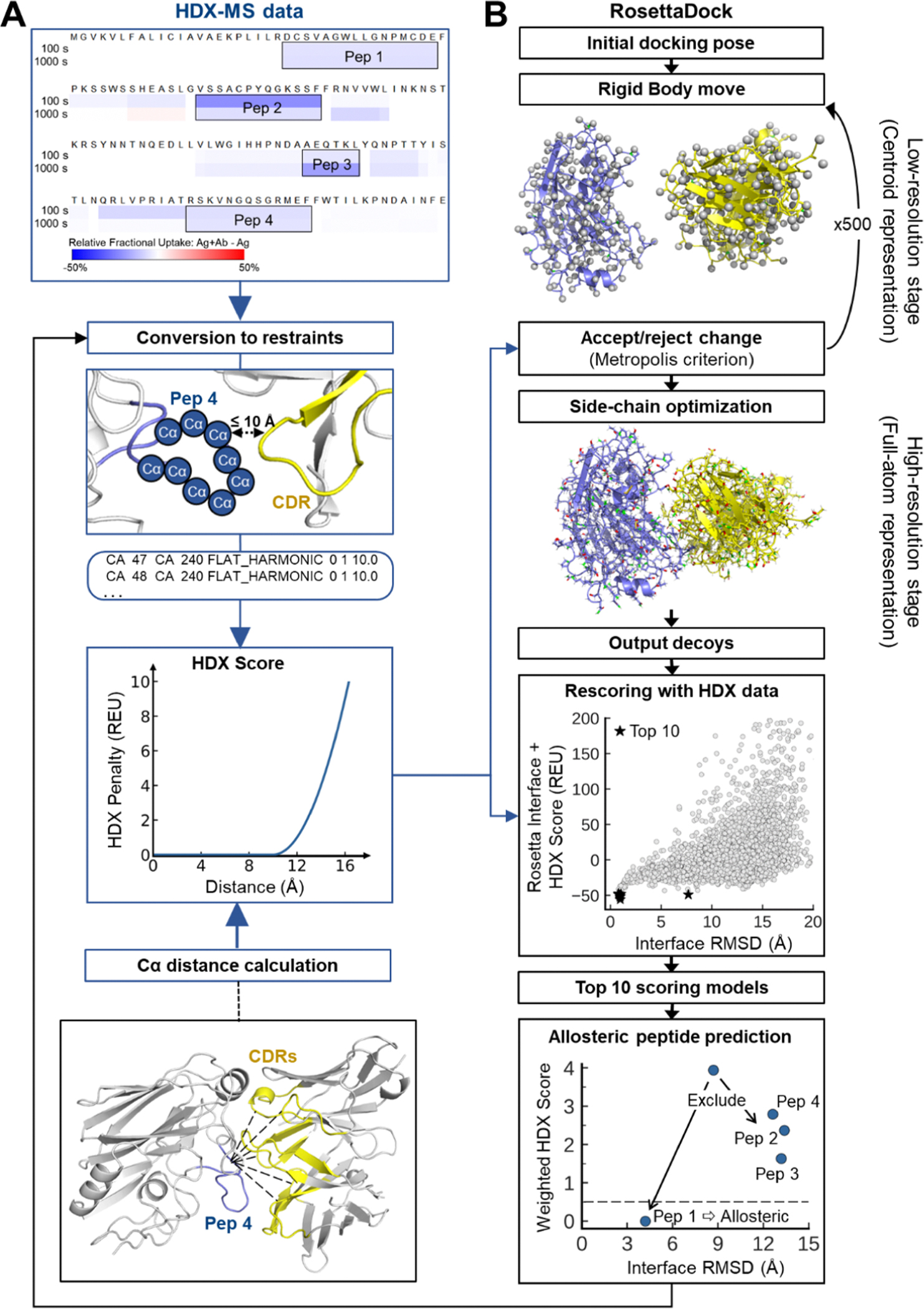
Workflow of integrative HDX-guided Ab-Ag docking in RosettaHDX. (A) A flowchart (in blue) illustrating the conversion of HDX-MS experimental data into restraints for docking. Each HDX-interacting peptide must have at least one Cα atom within 10 Å of a Cα atom in the CDRs. For each docking model, the shortest Cα-Cα distance between each HDX-interacting peptide and the CDRs is measured, and the HDX penalty score is then computed, as shown in the HDX score function plot. (B) For model generation, the HDX penalty score is used in the Metropolis algorithm to guide conformational sampling during the low-resolution stage of RosettaDock. For model selection, the HDX penalty score is combined with the Rosetta interface score to choose the top 10 final models. The HDX scores of these top 10 models serve as a predictive metric for identifying allosteric peptides (Section 2.8), which can then refine the restraint set for re-scoring and re-sampling. (For interpretation of the references to color in this figure legend, the reader is referred to the web version of this article.)

The key input from HDX-MS experiments for RosettaHDX is potential interacting peptides on the Ag (Fig. 1A). Since there is no fixed standard for assigning significant differences in HDX-MS, which can vary by experimental setup (Masson et al., 2019), the interpretation of HDX-interacting peptides is entrusted to the researchers conducting the experiments (see Bangaru et al., 2019; Dong et al., 2020; Garcia et al., 2023; Zhang et al., 2013 for published HDX results, and Methods for our unpublished in-house data). Each HDX-interacting peptide was implemented as a restraint with a penalty. We explored a range of HDX distance restraint parameters, from relaxed to stringent (Fig. S2, details in Methods). Our selected optimized restraint requires the Cα of at least one amino acid (excluding Proline) in an HDX-interacting peptide to be within 10 Å of any Cα in the Ab CDRs, reflecting the primary engagement sites of most Abs with Ags (Fig. 1A). While this setup is tailored to Ab-Ag docking, a similar approach can be extended to other systems where the interacting regions are known.

For each docking model, the shortest Cα distance between an HDX-interacting peptide and the CDRs is calculated and input to a flat harmonic function (see Eq. (1) in Methods) to yield the penalty (Fig. 1A). These scores are then aggregated across all HDX peptides in each model. Models that satisfy all HDX restraints receive a zero-penalty score, while deviations lead to higher scores.

### 2.3. Applying HDX restraints for sampling, scoring, and identifying allosteric peptides in RosettaHDX

RosettaDock operates in two phases: a low-resolution centroid phase to determine the optimal orientation of binding partners, followed by a high-resolution all-atom phase to refine docking positions and side-chain interactions (Gray et al., 2003a; Gray et al., 2003b; Jeliazkov et al., 2021; Marze et al., 2018). Since the low-resolution phase performs most of the global search for binding orientations, we implemented HDX distance restraints at this stage to maximize their impact on guiding model generation. Changes in penalty score influence the Monte Carlo sampling by either discouraging or encouraging the acceptance of new docking conformations (Fig. 1B).

Docking models generated by RosettaDock are typically evaluated using the Rosetta interface score (dG_separated), which estimates the cross-interface binding energy. The Rosetta interface score represents the energy difference between the bound and unbound states of interface-forming chains, essentially measuring the energy required to dissociate the complex. This calculation relies on the standard all-atom energy function in Rosetta, REF15, which is based on physical, empirical, statistical and knowledge-based score terms expressed in Rosetta Energy Units (REU) (Alford et al., 2017; Leman et al., 2020). To enhance model selection accuracy by selecting models in agreement with HDX-MS data, we combined the Rosetta interface score with an HDX-guided score term. The HDX score is the cumulative penalty for deviations from the HDX distance restraints outlined in the previous section, calculated across all HDX-interacting peptides (Eq. (1)). It rewards models that align with the HDX data, assigning more favorable, near-zero scores to those in strong agreement. In the final RosettaHDX scoring function, the HDX score was normalized, weighted by an optimized value of 4.5, and added to the initial Rosetta interface score, as described in Eqs. (2) and (3) in the Methods section.

Finally, our predictive metric, based on the top 10 docking models, uncovered allosteric peptides (peptides with decreased deuterium uptake, but at sites distant from true binding sites), enabling refinement of HDX restraints for rescoring and redocking to improve model accuracy (Fig. 1B).

### 2.4. HDX-MS restraints enhanced sampling of native-like docking conformations

**Fig. 2.**
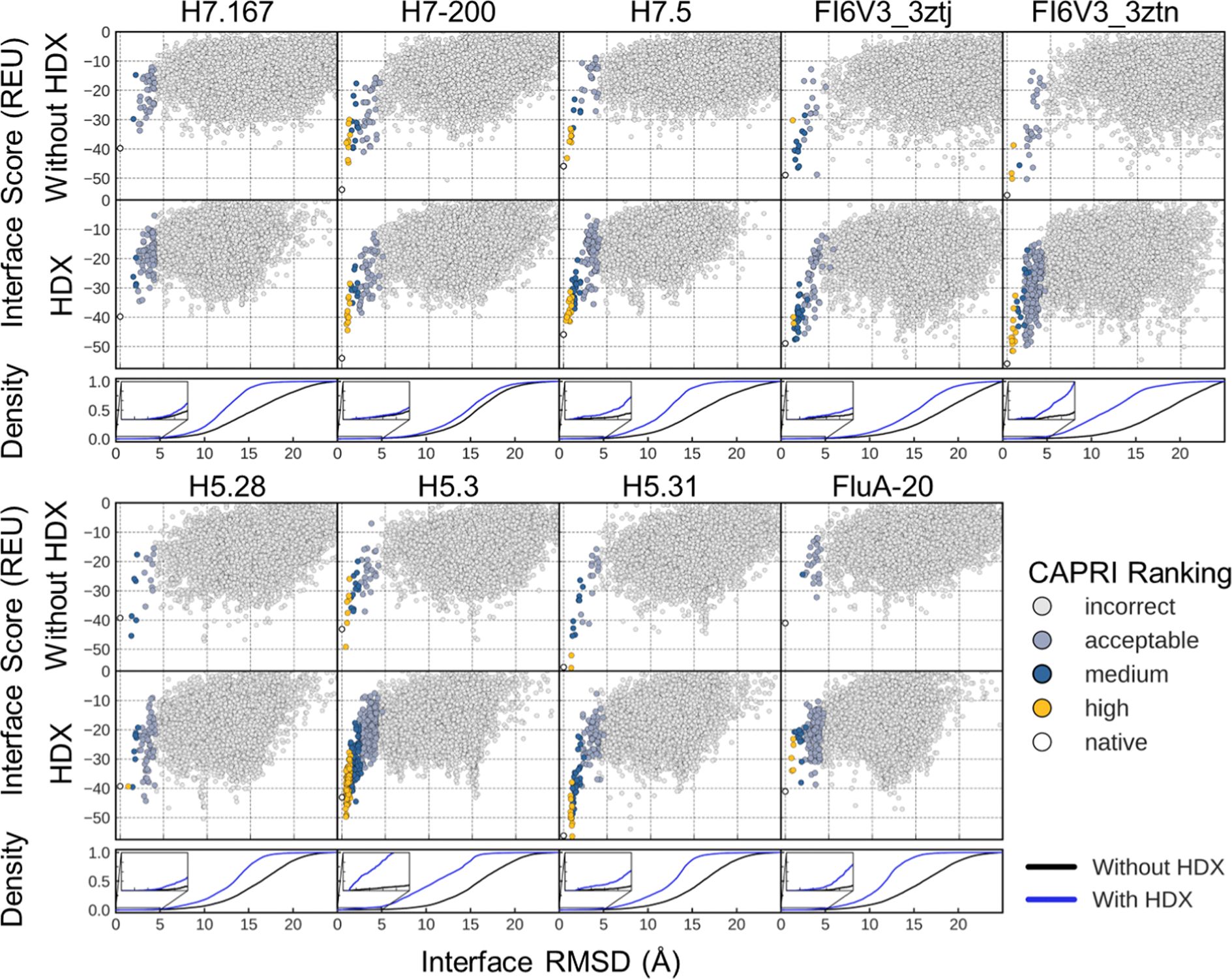
HDX-MS restraints enhanced sampling of native-like conformations. The figure displays Rosetta binding interface score versus interface RMSD plots, and cumulative fraction of models versus interface RMSD plots for 10,000 models generated using RosettaDock, with and without HDX distance restraints, across nine benchmark complexes. In the score-vs-iRMSD plots, each point represents one of the 10,000 models generated for each complex. Model accuracy is color-coded based on CAPRI criteria (Janin et al., 2003), as detailed in the legend: high (yellow), medium (dark blue), acceptable (light blue), and incorrect (light gray). The white circle marks the reference energy of the relaxed, bound crystal structure. The density plots display the cumulative fraction of models within the iRMSD range of 0 to 25 Å, comparing docking results without HDX distance restraints (black) to those with HDX restraints (blue). (For interpretation of the references to color in this figure legend, the reader is referred to the web version of this article.)

Starting from the bound crystal structure, we evaluated the efficiency of HDX distance restraints in guiding sampling toward native-like conformations. For each of the nine benchmark complexes, 10,000 models were generated with and without HDX restraints. The interface score versus interface root-mean square deviation (iRMSD) and density plots in Fig. 2 revealed an increased number of lower iRMSD (favorable) models among the 10,000 models generated using RosettaHDX for all nine complexes. The inclusion of HDX restraints resulted in a significant difference in the iRMSD-based cumulative distribution of generated models, as determined by the two-sample Kolmogorov-Smirnov test (p < 0.001). This was reflected in a leftward shift of the density curve for all complexes, indicating improved iRMSD values. The Kolmogorov-Smirnov statistic value, which ranges from 0 to 1 and reflects the extent of discrepancies between distributions, showed values between 0.14 (H7–200) and 0.65 (FI6V3_3ztn) across the nine complexes, with larger values indicating greater differences in iRMSD distribution between models docked with and without HDX restraints. Table S2 summarizes the average iRMSD of the top 1 % of models ranked by interface-RMSD to the native structure. Across all cases, HDX restraints resulted in lower iRMSD values for the best 1 % of models, with the average iRMSD significantly improving from 4.3 to 2.6 Å (see last row in Table S2 and Fig. S3A for statistical analysis). Of the nine complexes, three (H7.167, H7–200, and FI6V3_3ztn) have only one HDX-interacting peptide (Table S1), demonstrating that even a single HDX-interacting peptide was sufficient to benefit sampling.

Noticeably, HDX restraints greatly enhanced the sampling of native-like models. As shown in the density plot in Fig. 2, the addition of HDX restraints increased the population of models within the near-native iRMSD range of 0–5 Å for all nine complexes, with increases ranging from 1.4-fold (H7–200) to 10-fold (H5.3). In the interface score vs iRMSD plot in Fig. 2, models were classified into four quality categories—incorrect, acceptable, medium, and high-quality (and color-coded accordingly)—based on the CAPRI docking model quality criteria, which takes into account of the fraction of correctly predicted native contacts (fnat), ligand RMSD (L-RMSD), and interface RMSD (Janin et al., 2003) (Table 1). While RosettaDock alone generated near-native models (defined as models of acceptable quality or better) for all nine complexes, RosettaHDX on average produced approximately five times more high-quality models and four times more medium- and acceptable-quality models (Table S3). Furthermore, for H5.28 and FluA-20 complexes, HDX restraints enabled the generation of high-quality models (iRMSD ≤ 1 Å) that were not achieved with RosettaDock alone. Specifically, the best iRMSD model improved from 1.3 to 0.9 Å for H5.28 and from 2.0 to 0.7 Å for FluA-20 (Fig. S3B).

An important question we aimed to address in this work was how well allosteric peptides could be tolerated in an HDX dataset while still benefiting conformational search and model selection. Based on the known native crystal structures of our nine benchmark complexes, we classified that FluA-20, H5.28, H5.3, and H5.31 to each include one allosteric peptide in their HDX datasets. These allosteric peptides, while identified by HDX as showing significant changes in deuterium uptake, fail to engage in direct binding interactions and are located more than 10 Å from the interacting CDRs of the Ab in the native crystal structures. Specifically, H5.28 and H5.3 have one allosteric peptide out of three (peptide 84–95 and peptide 6–21, respectively), while H5.31 and FluA-20 have one out of four (peptide 83–95 and peptide 85–96, respectively) (Table S1; allosteric peptides are highlighted in green in Fig. S4). Here, in terms of conformational search, sampling improvements were observed in all four cases (Fig. 2, bottom row). This improvement suggested that, despite potential misguidance from allosteric peptides, the other true interacting peptides in the HDX dataset could compensate and still guide sampling toward near-native conformations. In other words, our current results indicated that an HDX dataset with up to 33 % false positives (e.g. one allosteric peptide out of three) can still be tolerated and positively impact Ab-Ag binding conformation search in RosettaHDX. Overall, in this section, we demonstrated that sampling was enhanced in RosettaHDX, even in the presence of allosteric peptides.

### 2.5. HDX-MS scoring function improved model section accuracy

Above, we tested the ability of an HDX-derived penalty term to guide model generation. Here, we investigated the potential of the HDX score to improve model selection in RosettaHDX. We anticipated that HDX scoring could enhance model selection in docking simulations that have not incorporated HDX restraints. Additionally, we aimed to determine whether HDX scoring could further differentiate model quality after HDX restraints were applied for sampling. To do this, we rescored the 10,000 existing models—generated with and without HDX restraints—using the combination of HDX score and Rosetta binding energy (Fig. S5 and Eqs. (2) and (3), details in the Methods section). Rescoring does not change models’ iRMSD values but only reevaluates their relative scores.

**Fig. 3.**
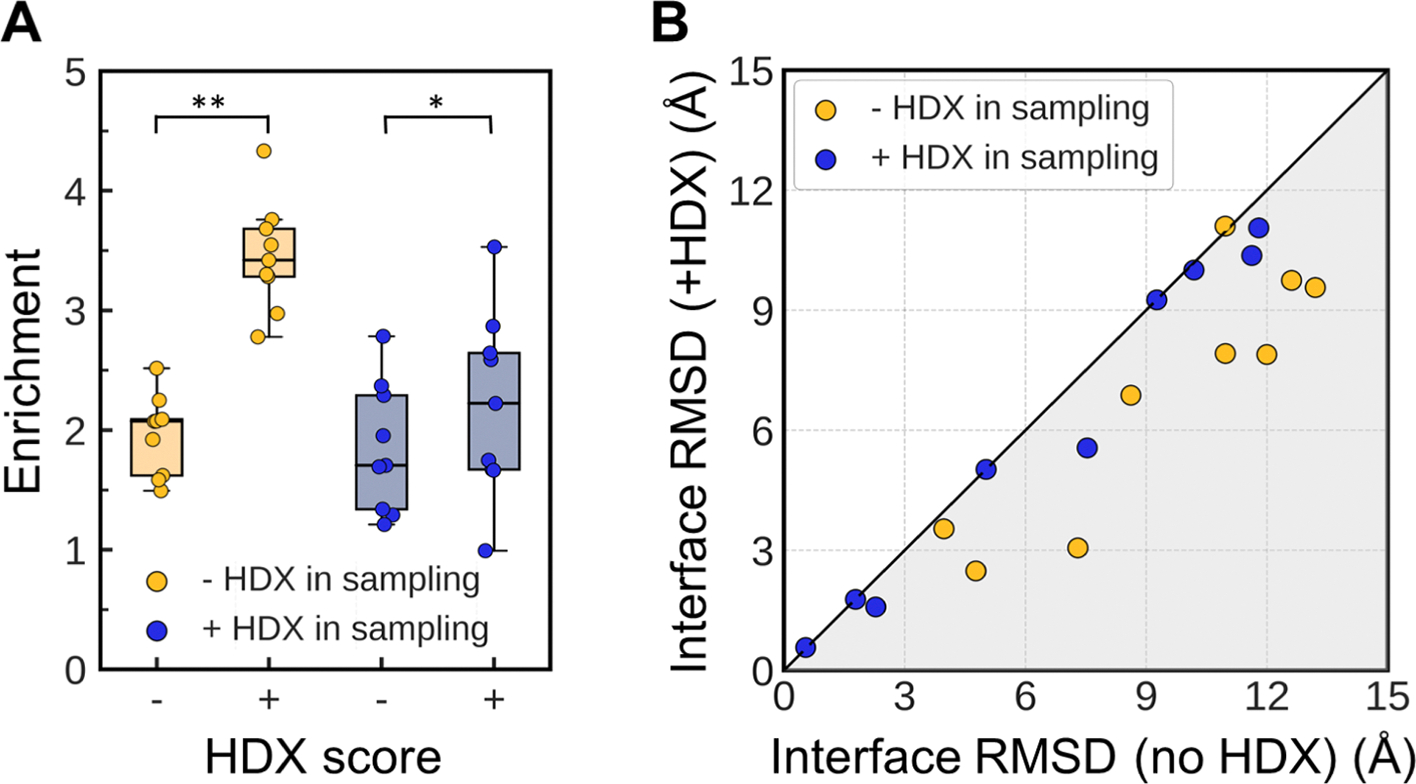
HDX scoring function improved docking model selection. Docking ensembles generated with and without HDX data during sampling are shown in blue and yellow, respectively. (A) Model enrichment was calculated when models were scored with Rosetta interface score alone and with the addition of the HDX score. The box-and-whisker plot displays whiskers extending to 1.5 times the interquartile range of the lower and upper quartiles. Statistical comparisons were assessed using a two-tailed Wilcoxon signed-rank test (n = 9, *p < 0.05, **p < 0.01). (B) Comparison of the average interface RMSD among the top 10 scoring models when scored with Rosetta interface score alone and with the addition of the HDX score. Gray area indicates iRMSD improvement of the top 10 scoring models with the addition of the HDX score. Complete scoring results are in Fig. S6. (For interpretation of the references to color in this figure legend, the reader is referred to the web version of this article.)

With our optimized recombined score, the average enrichment across the nine complexes increased significantly from 2.0 to 3.5 for models generated without HDX restraints and from 1.8 to 2.2 for models generated with HDX restraints (Fig. 3A). The top 10 models selected before and after HDX rescoring for each benchmark complex are depicted in Fig. S6, with their average iRMSDs shown in Fig. 3B, S3C, and Table S2. For models generated without HDX restraints, HDX rescoring reduced the top 10′s average iRMSD in 8/9 complexes, improving from 9.4 to 6.9 Å overall. For models generated with HDX restraints, 5/9 complexes showed a reduced average iRMSD of the top 10 models, with no worsening in the remaining 4 complexes (overall improvement from 6.7 to 6.2 Å). The number of near-native models in the top 10 selected by HDX score increased for 7/9 complexes generated without HDX restraints, and for 3/9 complexes generated with HDX restraints.

In summary, incorporating the HDX score into Rosetta’s energy function significantly improved the quality of selected models, with a greater impact on models generated without HDX restraints than on those generated with them. This outcome was expected, as the discriminative power of HDX score diminished when HDX data had already been applied during sampling.

### 2.6. HDX-MS data improved iRMSD of best scoring models when docking with Ab and Ag models

Initially, to establish and benchmark RosettaHDX, we performed redocking using subunit structures derived from the bound co-crystal structure. Here, we sought to evaluate the performance of our established method in a more realistic setting using predicted models of each subunit as docking inputs. Given AF’s accuracy in de novo structure prediction, we employed AF2 to generate Ag and Ab models. Prior to docking, AF-predicted models underwent AF’s built-in AMBER relaxation protocol (Hornak et al., 2006), a gradient descent minimization in the AMBER force field designed to remove stereochemical violations and physically impossible atom clashes (Jumper et al., 2021). These relaxed AF Ab and Ag models then underwent Rosetta relaxation, where backbone and side-chain torsion angles were optimized through multiple rounds of packing and minimization using Monte Carlo calculations (Conway et al., 2014). The five top-ranked AF Ab models were paired with the five top-ranked AF Ag models, creating an ensemble of 25 pre-docking starting conformations with diverse and biophysically plausible backbone conformations. The AF models were reasonably accurate, with all Ab models having heavy-chain complementarity-determining region 3 (HCDR3) RMSDs < 4.0 Å (averaging 2.3 Å) and all Ag models having RMSDs < 2.8 Å (averaging 1.3 Å). Using these models as input, we performed RosettaHDX following the previously described procedure, with HDX data applied to both sampling and scoring.

**Fig. 4.**
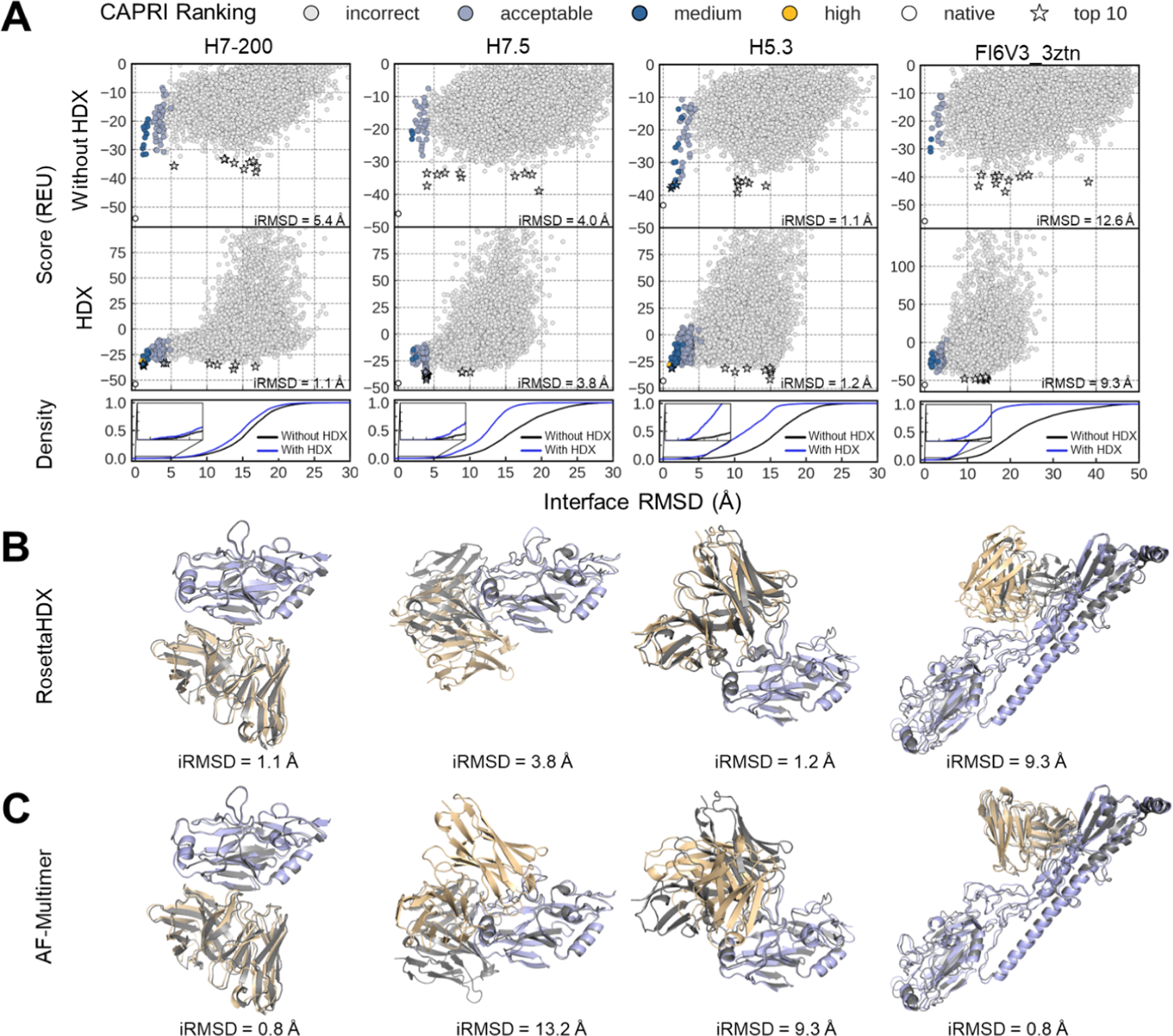
Docking results of four complexes in the benchmark set using AF models as docking input. **(A)** Score vs. iRMSD and cumulative fraction of model plots for 10,000 docked models generated for H7–200, H7.5, H5.3, and FI6V3_3ztn using AF models as docking input. Rosetta interface score vs iRMSD plots are shown for docking ensemble predicted without HDX. Combined HDX and Rosetta interface score vs iRMSD plots are shown for docking ensemble predicted with RosettaHDX. The combined score is less negative than the Rosetta interface score due to added positive HDX penalty score. The best iRMSD among the top 10 scoring models is indicated in the lower right corner of each plot. Model accuracy is color-coded based on CAPRI criteria (Janin et al., 2003), as detailed in the legend: high (yellow), medium (dark blue), acceptable (light blue), and incorrect (light gray). The white circle marks the reference energy of the relaxed, bound crystal structure. Top 10 scoring models are marked by star symbols and colored according to the CAPRI criteria. Density plots show cumulative fraction of models within the iRMSD range of 0–30 Å for H7–200, H7.5, and H5.3, and 0–50 Å for FI6V3_3ztn, comparing docking results without HDX distance restraints (black) and with HDX restraints (blue). Full results panel for the remaining 6 benchmark complexes is shown in Fig. S7 and S9A. **(B)** Best iRMSD model (among the top 10 scoring models) generated by RosettaHDX (Ab: light yellow, Ag: light blue) superimposed on the native crystal structure (dark gray), with iRMSDs listed below the alignments. **(C)** Best iRMSD model (among the top 10 AF ranked models) generated by AF-Multimer (Ab: light yellow, Ag: light blue) superimposed on the native crystal structure (dark gray), with iRMSDs listed below the alignments. (For interpretation of the references to color in this figure legend, the reader is referred to the web version of this article.)

As a comparison to state-of-the-art methods, we also used AF-Multimer to predict the complete structure of the complexes in our benchmark set. A successful docking attempt is defined as having a model of acceptable quality or better in the top 10 scoring models (Janin et al., 2003). Fig. 4A summarizes the docking performance of RosettaHDX compared to Rosetta alone for 4/9 complexes, where successful docking attempts were made by either RosettaHDX or AF-Multimer. The score versus iRMSD plots illustrated the quality of the top 10 scoring models and the best iRMSD achieved among the top 10 for each complex, both with and without HDX data.

In previous redocking experiments using inputs derived from co-crystal structures, RosettaHDX successfully predicted 7/9 complexes, with 3/7 showing an increase in near-native models within the top 10 compared to Rosetta alone (Fig. S6A and Table S3). When docking with AF models, we observed fewer successful predictions as expected. Using Rosetta alone, only 1/9 complexes (H5.3) was successfully predicted, with a best iRMSD of 1.1 Å among the top 10 selected models. With the addition of HDX data, RosettaHDX improved docking success, with 3/9 complexes (H5.3, H7–200, and H7.5) successfully predicted. Notably, RosettaHDX improved the minimum iRMSD among the top 10 scoring models from 5.4 to 1.1 Å for H7–200 and from 4.0 to 3.8 Å for H7.5, both of which had failed with RosettaDock alone. Although RosettaHDX predictions for FI6V3_3ztn and five other complexes were not successful (investigated in Section 2.7), RosettaHDX still improved the average iRMSD of their top 10 scoring models by more than 1.5 Å for all six complexes, indicating strong improvement in docking accuracy, with FI6V3_3ztn and FI6V3_3ztj both showing improvements of over 6 Å (Table S4).

Overall, we demonstrated that RosettaHDX, initially established and refined using crystal structures as docking input, continued to improve docking accuracy when using AF models as input. The incorporation of HDX consistently enhanced sampling (Figs. S7, S8, and Table S4) and improved model selection quality (Fig. S9, S10, and Table S4), with a detailed analysis provided in the SI. Fig. 4B shows the best iRMSD model among the top 10 scoring for four complexes—H7–200, H7.5, H5.3, and FI6V3_3ztn—generated by RosettaHDX, aligned to their native crystal structures. Fig. 4C presents the best iRMSD model among the top 10 AF-ranked models generated by AF-Multimer for these same complexes, with iRMSDs of 0.8 Å, 12.3 Å, 9.3 Å, and 0.8 Å, respectively. 2/9 benchmark complexes—H7–200 and FluA-20—were successfully predicted by AF-Multimer, with one overlapping a success case from RosettaHDX. Furthermore, there were complexes that RosettaHDX successfully predicted but AF-Multimer failed to (H7–200 and H5.3), and vice versa (FI6V3_3ztn). This indicates that both methods have their own valuable contributions and can serve as complementary approaches for docking Ab-Ag complexes.

### 2.7. Unsuccessful docking predictions were due to scoring failure in the initial Rosetta score without HDX data

Previously, we established that HDX improved docking performance in a realistic docking scenario, even for the six complexes that did not meet the docking success criteria. We suspect that the failure of these six complexes was largely due to the lack of accuracy in the initial Rosetta interface score without HDX data. Although near-native models were generated for all benchmark complexes (Fig. 4A and S9), with a 3.6-fold increase in number when HDX data was included (Table S3), these models were not selected in the top 10 for these six complexes, indicating a scoring issue.

Fig. S9A displays the quality of the top 10 models selected from the docking ensemble generated with HDX restraints, using the initial Rosetta interface score versus the combined HDX and Rosetta score. In the three successful cases (H5.3, H7–200, and H7.5), the initial Rosetta score distribution was close-to-accurate to include near-native models in the top 10, which the HDX score further enhanced. In the six unsuccessful complexes, however, the initial Rosetta score distribution without HDX data lacked sufficient accuracy (no near-native model selected among the top 10). Therefore, even though HDX score did improve model selection quality (Fig. S10), near-native models still failed to be included in the top 10 for these complexes. This is understandable, because the sparse nature of HDX data means that HDX scoring alone cannot reliably distinguish non-native models from native ones; it mainly adds distinguishing power by flagging models that violate HDX restraints. Consequently, for structural modeling with sparse data, the successful selection of native-like models depends heavily on the initial accuracy of the Rosetta score distribution, a limitation previously acknowledged in Marzolf et al., 2021. Overall, despite the fact that RosettaHDX could not select native-like structures to be in the top 10 for all complexes, we demonstrated that this was likely due to inaccuracies in the initial Rosetta interface score, and that the incorporation of HDX was never detrimental and consistently improved docking performance over Rosetta alone.

### 2.8. Predictive metric allows identification of HDX allosteric peptides

In previous sections, we optimized our HDX restraint settings and scoring weights to minimize potential misleading effects of allosteric peptides on model generation and selection. Here, we investigated whether we could develop a metric based on RosettaHDX docking results to identify allosteric peptides within an HDX dataset, thereby enhancing the effectiveness of HDX score in distinguishing model quality. To this end, we examined the average weighted HDX score of the top 10 RosettaHDX models (selected by the combined Rosetta interface and HDX score). Assuming that these top scoring models are native-like, their HDX penalty scores should be close to 0 if all restrained peptides belong to the true binding interface. Conversely, their HDX penalty scores would be higher if non-binding-site peptides are restrained. Further details on the rationale behind developing this metric are provided in the SI and Fig. S11.

**Fig. 5.**
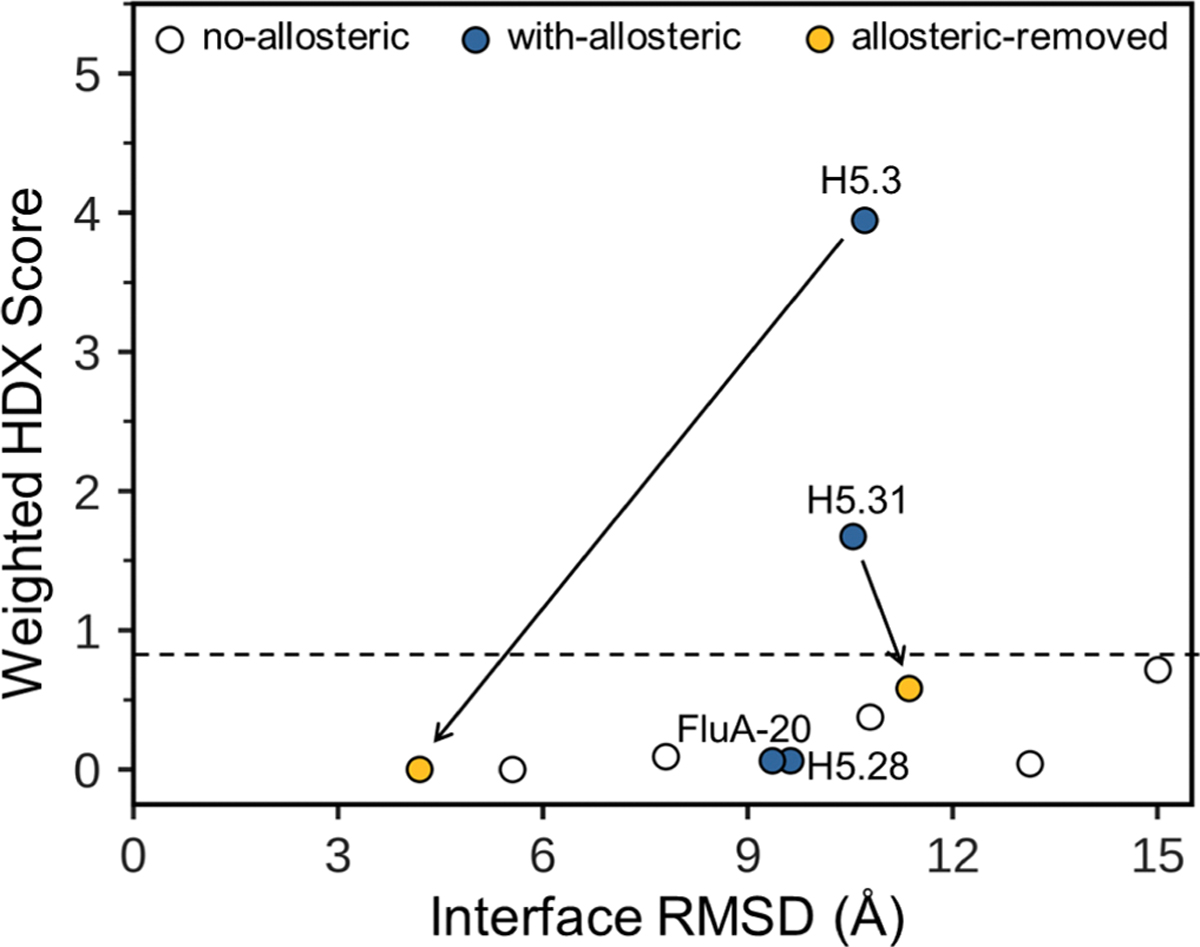
Predictive metric for HDX allosteric peptides. Average weighted HDX score versus interface RMSD of the top 10 scoring models generated by RosettaHDX starting with AF models as docking input. The dashed line represents the prediction cutoff of 0.75, such that complexes with an average weighted HDX score above the line are predicted to contain allosteric peptides in their HDX dataset. Each point represents the top 10 scoring models of each benchmark complex. Complexes without allosteric peptides, with allosteric peptides, and with allosteric peptides identified and excluded are colored white, blue, and yellow, respectively. Arrows indicate the change in the selection of the top 10 scoring models for H5.3 and H5.31 (with corresponding changes in weighted HDX score and interface RMSD) when the allosteric peptide was identified and excluded from scoring. (For interpretation of the references to color in this figure legend, the reader is referred to the web version of this article.)

Fig. 5 illustrates the average weighted HDX score versus average iRMSD of the top 10 scoring models for each benchmark complex docked with AF model inputs. The highest average weighted HDX score among the 5 complexes without allosteric peptides was around 0.75 (Fig. 5 and Table S5). Based on this, we set a predictive threshold of > 0.75 to maximize the sensitivity of our metric for detecting allosteric peptides. Using this threshold, all 5 benchmark complexes without HDX allosteric peptides were correctly categorized, and 2/4 complexes containing an allosteric peptide—H5.3 and H5.31—were correctly flagged.

The predictive metric failed to detect allosteric peptides in H5.28 and FluA-20. This was because its distinguishing power relied on the assumption that the top 10 scoring models would be predominantly near-native and would not satisfy restraints from allosteric peptides. Essentially, we relied on the Rosetta score to favor poses that did not meet allosteric restraints over those that did, expecting this effect to outweigh the influence of the HDX score favoring the opposite, thereby allowing the combined Rosetta and HDX score to select near-native models in the top 10. However, in cases like H5.28 and FluA-20, non-native poses that satisfied the allosteric peptide were also favored by Rosetta, resulting in a low, favorable average weighted HDX score for the top 10 scoring models (Fig. S9A), causing the metric to fail in detecting allosteric peptides. This outcome highlighted that our metric can only effectively predict allosteric peptides in cases where the assumption underlying its distinguishing power is met.

For the two successfully flagged complexes, H5.3 and H5.31, we further identified the most likely allosteric peptide within the HDX set. This was achieved by sequentially excluding each HDX peptide from the HDX score and selecting a new set of top 10 scoring models using the updated HDX scores combined with Rosetta interface score. The peptide whose exclusion led to the lowest average HDX score of the new top 10 was predicted to be the allosteric peptide. If the new average HDX score remains above the threshold, it indicates the potential presence of more than one allosteric peptide in the HDX dataset, and the process can be repeated to identify additional allosteric peptides. For both H5.3 and H5.31, our approach accurately identified the actual allosteric peptides, with updated HDX scores of 0.58 and 0, respectively; both were below the threshold, indicating no further allosteric peptides (Fig. 5 and Table S5). Among the finally selected top 10 models based on the updated allosteric-peptide-excluded HDX scores combined with Rosetta score, 9/10 models for H5.3 and 8/10 models for H5.31 had the allosteric peptides positioned more than 10 Å away from the interacting CDRs of the Abs, as ideally expected. The performance of our predictive metric for docking complexes starting from crystal structures is shown in Fig. S12 (detailed analysis in the SI).

The advantage of our predictive metrics could be further maximized by redocking with an updated set of HDX restraints that excludes the allosteric peptides (Fig. 1). While the predictive metric with rescoring only has performed well with single allosteric peptides in our benchmark complexes, if the new average HDX score suggests multiple allosteric peptides, we recommend combining redocking and rescoring to enhance the accuracy of the docked models.

Overall, our predictive metric proved to be a powerful tool for detecting allosteric peptides when this information was unknown. Once the identified allosteric peptide was excluded from the HDX score, and assuming the initial Rosetta binding score was sufficiently accurate as discussed in Section 2.7, the updated HDX score further enriched native-like models among the top 10 scoring. For instance, in H5.3, the new HDX score increased the number of native-like models selected among the top 10 scoring models from 2 to 7, significantly reducing the average iRMSD of the top 10 scoring from 10.4 to 4.2 Å. To our knowledge, we are the first to develop a predictive metric for detecting HDX allosteric peptides from docking experiment results.

### 2.9. Evaluation of alternative scoring methods and implications for RosettaHDX

To evaluate whether docking model accuracy could be assessed better by other scoring methods than Rosetta interface score, we rescored our docking models generated with HDX restraints with HADDOCK (Dominguez et al., 2003a) and ZDOCK (ZRANK being the corresponding score) (Pierce and Weng, 2008; Pierce et al., 2011), two of the most widely used docking programs in the field, and AF2Rank (Roney and Ovchinnikov, 2022), a state-of-the-art machine-learning-based model that evaluates accuracy using AF confidence scores. This rescoring experiment aimed to inform potential future directions for integrating these scores with the HDX score to improve top 10 model selections in RosettaHDX.

**Fig. 6.**
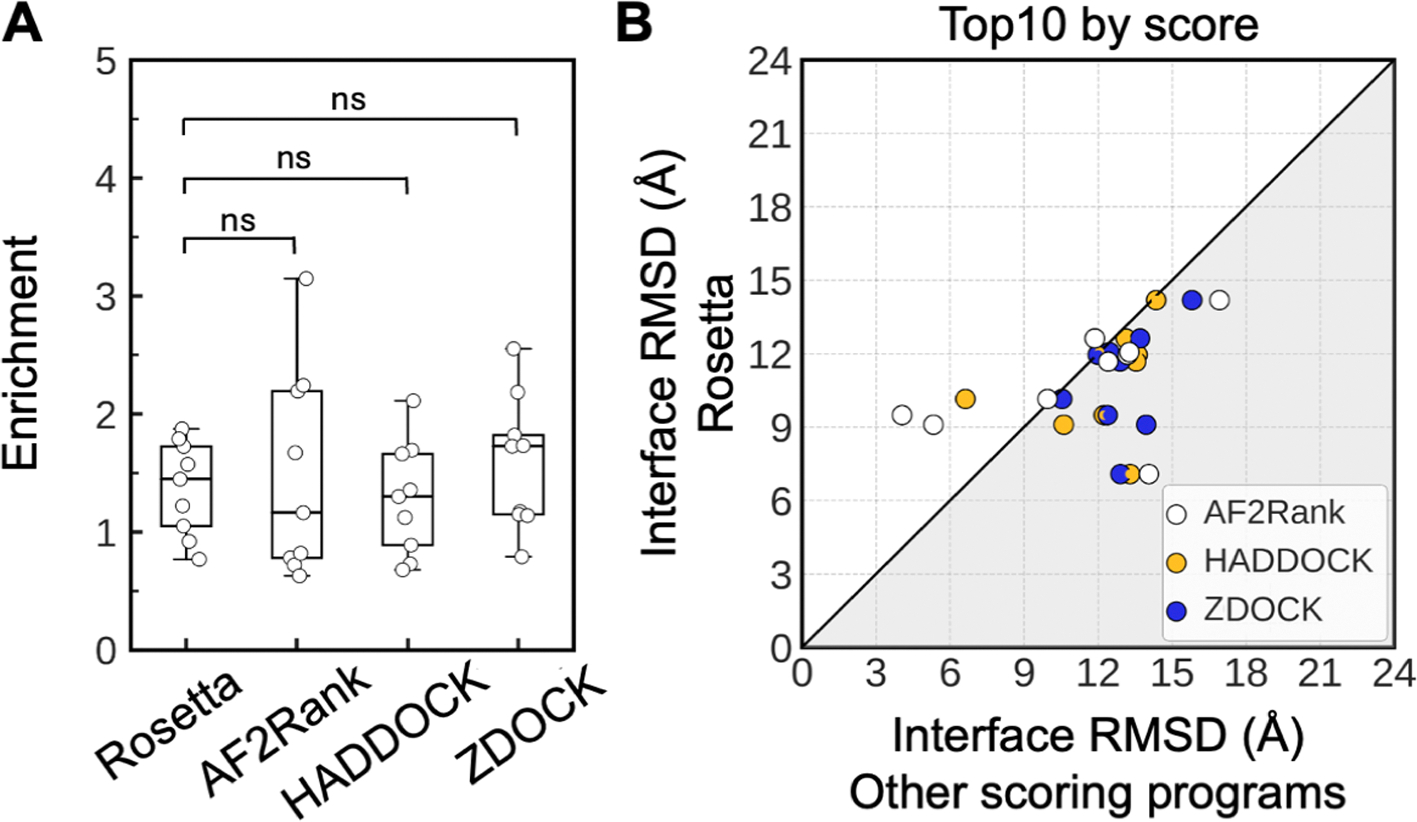
Comparison of different scoring programs with Rosetta in assessing docking models accuracy. Docking models of nine benchmark complexes generated with HDX restraints using AF individual subunit structures, originally scored with Rosetta interface score, were rescored with AF2Rank, HADDOCK, and ZDOCK. **(A)** Model enrichment was calculated when models were scored with Rosetta (interface score), AF2Rank (AF confidence score), HADDOCK (HADDOCK score), and ZDOCK (ZRANK score). The box-and-whisker plot displays whiskers extending to 1.5 times the interquartile range of the lower and upper quartiles. Statistical comparisons were assessed using a two-tailed Wilcoxon signed-rank test (n = 9, ns – not significant). **(B)** Comparison of the average interface RMSD among the top 10 scoring models when scored with Rosetta interface score versus other scoring programs (AF2Rank in white, HADDOCK in yellow, and ZDOCK in blue). Gray area indicates iRMSD improvement of the top 10 scoring models when scored with Rosetta. Complete scoring results are shown in Fig. S13, S14, and S15. (For interpretation of the references to color in this figure legend, the reader is referred to the web version of this article.)

The enrichment of HADDOCK, ZDOCK, and AF2Rank scoring across nine benchmark complexes were not significantly different from Rosetta interface score (Fig. 6A). However, Rosetta interface score selected top 10 models with lower average iRMSD than HADDOCK for 8/9 complexes, ZDOCK for 9/9 complexes, and AF2Rank for 5/9 complexes (Fig. 6B). HADDOCK and ZDOCK both identified one near-native models in the top 10 for H7–200 (Figs. S13 and S14). AF2Rank identified six near-natives for H7–200 and seven near-natives for H5.3 (Fig. S15). In comparison, Rosetta interface score identified near-natives in the top 10 for H7–200 (three near-natives), H5.3 (three near-natives), and H7.5 (five near-natives) (Fig. S9A).

While none of the methods successfully identified near-native models for the majority of cases, the Rosetta scoring function performed reasonably well compared to these other widely used methods, including the current state-of-the-art machine-learning-based AF2Rank program. This aligns with previous findings (Yin et al., 2022), which demonstrated that Rosetta interface score performed equivalently to ZRANK and AF confidence scores in classifying model accuracy in a benchmark of 149 complexes, with a slight advantage in distinguishing incorrect from high-quality models.

Interestingly, in this rescoring experiment, all programs successfully identified near-native models for the same specific complexes. Specifically, all methods succeeded with H7–200, and both AF2Rank and Rosetta succeeded with H5.3. As these programs failed on many of the same cases and succeeded on a limited subset, this suggests that combining these scoring functions is unlikely to significantly improve the overall success. However, our small benchmark dataset limits the ability to draw this conclusion with certainty, and further testing on additional complexes is needed, as respective HDX data becomes available.

That said, AF2Rank scoring results appear promising and worth exploring as a future direction for improving RosettaHDX. For 4/9 complexes, AF2Rank achieved equivalent or better average iRMSD of top 10 models compared to Rosetta. While both AF2Rank and Rosetta succeeded with H7–200 and H5.3, AF2Rank identified almost more than twice as many near-natives in the top 10 compared to Rosetta. Additionally, because AF confidence score leverages machine-learning-based pairwise error prediction to estimate model confidence (Evans et al., 2022), incorporating it (in place of Rosetta interface score) with HDX score would provide an orthogonal model evaluation perspective compared to the physics- and knowledge-based potential scoring that was relied on throughout the entire model generation process in RosettaHDX protocol. Overall, AF confidence score demonstrated promising potential and merits further investigation for integration with HDX score in future iterations of RosettaHDX.

## 3. Conclusions and future directions

HDX-MS is an increasingly adopted tool, well-suited for rapidly characterizing Ab-Ag interactions. In this study, we presented a strategy for incorporating HDX-MS data into computational docking. Our implementation of HDX-MS-derived restraints in RosettaDock, RosettaHDX, improved docking accuracy by enhancing both sampling and scoring. Using unbound AF Ag and Ab models as docking inputs, HDX-MS restraints successfully generated near-native models for all nine benchmark complexes and significantly increased their numbers by an average of 3.6-fold. In 2/9 cases, HDX restraints enabled the sampling of high-quality docking models that were not achievable without HDX data. Ultimately, RosettaHDX—applying HDX restraints in both sampling and scoring, and refining them for complexes with allosteric peptides—improved the average iRMSD of the top 10 selected models across all nine complexes, achieving an average improvement of 4.4 Å, with a minimum improvement of 1.8 Å.

Accurate prediction of Ab-Ag complexes has always been inherently challenging (Dapkūnas et al., 2021; Lee et al., 2022; Sela-Culang et al., 2013a). AF-Multimer, the state-of-the-art machine learning method for protein complex structure prediction, achieved a 20 % success rate for Ab-Ag complexes the original version, and only improved to over 30 % in version 2.3 in a recent benchmark (Yin and Pierce, 2024; Yin et al., 2022). This difficulty was also highlighted in the most recent CASP15 evaluation, which reported poor overall performance in the nanobody/Ab-Ag category, with only 1/5 nanobody-Ag complex well predicted and 1/3 Ab-Ag complexes predicted with acceptable to medium quality among all competitors (Lensink et al., 2023). Using the stringent CAPRI success criteria employed in CASP—which requires not only generating near-native models but also selecting them in the top-scoring ensembles—RosettaHDX was successful in 3/9 cases. In comparison, the state-of-the-art AF-Multimer met the docking success criteria in 2/9 cases in our benchmark set, with one success overlapping with RosettaHDX.

While RosettaHDX did not achieve perfect success for all complexes based on CAPRI criteria, it consistently improved docking model quality compared to the original RosettaDock, without being detrimental. To our knowledge, no other platform has benchmarked HDX-MS data in docking. This makes our study the first to optimize and calibrate restraint and scoring settings specifically for HDX data and to assess its impact on docking performance. We also explored the prerequisites and tolerances for an HDX dataset to improve docking performance, demonstrating effectiveness with as few as one interacting peptide or up to 33 % false-positive data points (e.g., one allosteric peptide out of three HDX peptides). The framework established for RosettaHDX could be extended to broader protein–protein docking scenarios by applying HDX-derived restraints to binding partners more generally, rather than limiting them to CDR regions. Finally, we are the first to our knowledge to devise a predictive metric to identify allosteric peptides based on docking results with HDX data. To sum up, with HDX-MS data acquisition taking as little as one week per epitope mapping experiment, RosettaHDX enables more reliable predictions of Ab-Ag complexes in relatively short timeframes. This makes our approach a valuable tool for high-throughput characterization of Ab-Ag complexes in structure-based therapeutic and vaccine development.

RosettaHDX has its limitations and areas for improvement. One key limitation is its ability to select near-native models within the top-scoring ensemble, which is constrained by the accuracy of the original Rosetta interface scoring function. We tested several widely used scoring programs as potential alternatives to combine with the HDX score to improve model selection in the future. Preliminary results with AF confidence score (pTM + iPTM) are promising and merit further investigation with additional benchmark complexes. Additionally, interface pLDDT, an AF-derived metric providing residue-level confidence scores over interface residues, was recently suggested to outperform the default AF confidence score for Ab-Ag models (Yin and Pierce, 2024) and could be explored for integration into RosettaHDX.

Second, our benchmark dataset is relatively small due to the limited availability of Ab-Ag complexes with both high-resolution crystal structures and HDX-MS data. Although HDX-MS has become the most widely adopted for MS-based epitope mapping in recent years, (James et al., 2021; Konermann and Scrosati, 2024), high-quality benchmark complexes remain scarce, as HDX-MS is often performed as a quick screen for complexes unsuitable for X-ray crystallography or when resources for crystallography are limited. As a result, our benchmark datasets are influenza-related, driven by availability rather than design. However, the general strategy and implementation of RosettaHDX did not rely on influenza-specific information, suggesting broader applicability to other Ab-Ag systems. The limitation of having small HDX-MS training datasets is not uncommon; for instance, methods developed for HDX data like AI-driven back-exchange correction or protection factor predictions, were trained on datasets with only 4–5 proteins (Salmas and Borysik, 2021; Salmas and Borysik, 2024; Salmas et al., 2023). Similarly, computational benchmark studies incorporating other sparse experimental data techniques have relied on datasets with 2–9 proteins (Aprahamian et al., 2018; Biehn et al., 2022; del Alamo et al., 2021; Drake et al., 2022; Seffernick et al., 2019). We anticipate improvements, especially in distinguishing allosteric peptides as future iterations of RosettaHDX benefit from additional benchmark datasets containing a higher proportion of allosteric peptides.

As RosettaHDX is built upon a semi-flexible docking protocol, its performance could benefit from more extensive sampling of backbone conformations, particularly in cases involving significant binding-induced conformational changes. Future efforts could explore strategies such as using molecular dynamics (MD) simulations to increase conformational flexibility in the starting ensembles, especially in the HCDR3 regions of Abs (Fernández-Quintero et al., 2019) or adopting a more aggressive backbone sampling approach during Rosetta’s low-resolution docking stage (Harmalkar et al., 2022). Lastly, RosettaHDX works with peptide-level HDX data. Recent advancements in automated tools that infer residue-resolved exchange rates from peptide-level HDX data (Crook and Gittens, 2024; Salmas and Borysik, 2021; Smit et al., 2021) could enhance the resolution and interpretative power of HDX input in RosettaHDX. Early-stage AI-driven HDX tools, such as those predicting protein dynamics from sequence data (Yu et al., 2023) or using optimized HDX-MS data to predict protein secondary structure (Salmas et al., 2023) provide a promising foundation for leveraging AI to model protein conformations with HDX-MS data (Smit et al., 2021). Looking ahead, advancements in AI and the availability of larger benchmark datasets offer an exciting opportunity to integrate the principles and foundations of RosettaHDX into AI-based structure prediction workflows.

## 4. Methods

A tutorial on using RosettaHDX – RosettaDock with HDX-MS restraints, including a summary of necessary files and command lines, can be found in the Protocol Capture in the SI document accompanying this publication. Rosetta version 3.13 was used throughout the study. Rosetta is freely accessible for academia at the address: https://www.rosettacommons.org.

### 4.1. Benchmark set of Ab-Ag complexes with HDX-MS data

Our benchmark set comprises nine Ab-Ag complexes targeting influenza hemagglutinin, with available co-crystal structures and HDX-MS data. These complexes are: H7.167 Ab and H7–200 Ab, both bind to HA A/Shanghai/02/2013 (PDB 5V2A and 6UIG); H7.5 Ab binds to HA A/New York/107/2003 (PDB 6MLM); FI6V3 Ab binds to HA A/Aichi/2/1968 (H3) (PDB 3ZTJ) and to HA A/California/04/2009 (H1) (PDB 3ZTN); H5.28, H5.3, and H5.31 Ab bind to HA A/Vietnam/32/2004 (PDB 6P3S, 4XNQ, and 6P3R); and FluA-20 Ab binds to HA A/Solomon Islands/3/2006 (PDB 6OC3). The interacting HDX peptides were derived from HDX experiments reported by Sheng-Li and colleagues, Kelly Lee and colleagues (Bangaru et al., 2019; Dong et al., 2020; Garcia et al., 2023; Zhang et al., 2013), and our unpublished in-house studies.

For the FluA-20 and H7.5 complexes, HDX experiments were performed on different strains than those in the crystal structures (Table S1 and Fig. S1). Nevertheless, given that the binding sites of these Abs are similar across both strains (Bangaru et al., 2019; Thornburg et al., 2016), we applied docking restraints to the crystal HA strain based on homologous regions of the HDX interacting peptides. A comprehensive summary of the protein complexes in our benchmark set and their HDX results is detailed in Table S1, and our corresponding unpublished HDX results are presented in Fig. S1.

Among all the complexes, HDX experiments were conducted using the monomeric head of HA1, except for the FI6V3 complexes. In the case of FI6V3 Ab, both the HDX experiments and the co-crystal structures utilized the full-length HA, which includes both HA1 and HA2 subunits (Garcia et al., 2023). Consequently, docking experiments for FI6V3 utilized the full-length HA, while those for the other complexes used only the HA1 monomeric head.

### 4.2. HDX-MS experiment

In-house HDX-MS was performed to map the epitopes of H7.5, H5.3, H5.31, and FluA-20, with HA and Fabs prepared at a concentration of 10 pmol/μL. Labeling occurred in PBS pH 7.4 in D_2_O at 20 °C for 100 s and 1000 s. The reaction was quenched in 4 M guanidine/HCl, 100 mM tris (2-carboxyethyl) phosphine to a pH of 2.3, 0 °C. Automated HDX incubations, quenches, and injections were performed using an HDX-specialized nano-ACQUITY ultraperformance liquid chromatography (UPLC) system coupled to a Xevo G2-XS mass spectrometer. Online digestion was performed at 15 °C using an immobilized-pepsin column with generated peptides immediately trapped at 0 °C on a VanGuard BEH C18 1.7 μm guard column. Peptides were eluted using 5 %–35 % acetonitrile, 0.1 % formic acid in H2O, and separated on a ACQUITY UPLC BEH C18 1.7 μm, 1 mm × 100 mm column with data being acquired using an MSe data independent analysis (DIA) strategy. Peptide identification was performed using Waters ProteinLynx Global Server 3.0.3 software and deuterium uptake was calculated using DynamX 3.0 soſtware. Potential interacting peptides were defined as those having at least one time point with a relative deuterium uptake difference of ≥ 0.5 Da ± 3.0 standard deviations between Fab-bound and unbound HA, with a cumulative difference of > 1.1 Da over the entire exposure time (Houde et al., 2011).

### 4.3. Model generation

Docking simulations require all-atom subunit structures as input to predict complex structures. In this study, we conducted two separate sets of docking experiments utilizing input structures obtained from different methods. Initially, to establish and benchmark RosettaHDX, we derived individual structures of each docking partner from bound crystal structures to perform a redocking study. Subsequently, to evaluate performance improvements of our established method under a more realistic prediction protocol, we initiated docking using AF models of Ab and Ag. Before docking, both the Ab and Ag were individually refined to resolve potentially unfavorable geometries or clashes using Rosetta FastRelax (Tyka et al., 2011). The docking protocol with RosettaDock was consistent across different types of input structures.

To establish the baseline performance of RosettaDock’s global docking without HDX-MS experimental knowledge, 10,000 models were generated for each of the nine benchmark complexes (Gray et al., 2003a; Jeliazkov et al., 2021; Marze et al., 2018). For experiments starting with the co-crystal structure, initial conformations were generated by displacing the Ab and Ag by 10 Å and rotating the Ag by 60° to scramble the interface. For consistency, experiments starting from AF Ab and Ag models first aligned the unbound models to the bound crystal structure before similarly disturbing the interface. Once the interface was scrambled, the position and orientation of the Ab were randomized around the Ag at the start of each simulation, using the –randomize2 flag in the RosettaDock protocol. This was followed by additional Gaussian random perturbations of 3 Å and 8° to create varied starting states and facilitate extensive local searching. In this low-resolution stage, models were evaluated using centroid-based energy functions, followed by REF15 scoring function in the high-resolution, full-atom refinement stage (Alford et al., 2017). The final ten docking models with the lowest (most favorable) Rosetta interface scores were selected as the top 10 predicted structures.

In RosettaHDX, distance restraints derived from potential interacting HDX peptides were incorporated into the low-resolution docking stage of RosettaDock, with all other settings unchanged. The HDX restraint penalty was combined with the Rosetta centroid score at a 1:1 ratio to guide conformational search. Binding conformations that violated HDX constraints were penalized (Eq. (1)), thereby discouraging their acceptance during the sampling process. A total of 10,000 models were generated, and the final models were selected based on the combined HDX and Rosetta interface scores.

### 4.4. Establishing HDX-MS distance restraints for Ab-Ag docking

We tested multiple HDX-MS distance restraint parameters. The most lenient setting required the Cα of at least one amino acid (excluding Proline) in each HDX-interacting peptide on the Ag to be within 10 Å of any Cα in the Ab CDRs. More stringent settings increased the required number of interface residues in each HDX peptide from 1 to 2 to 3 and reduced the Cα–Cα threshold distance from 10 Å to 8 Å to 5 Å. To assess each setting’s ability to distinguish near-native models from poor ones, we calculated the HDX penalty for 10,000 models generated without HDX guidance for each benchmark complex. Effective restraints would penalize poor models while sparing native-like ones.

For each HDX peptide, the shortest model-predicted Cα–Cα distance to the CDRs was calculated and input into a flat harmonic penalty function to compute the HDX penalty.


(1) $$f(x)=\left\{\begin{array}{c} 0\;\mathrm{for}\;x\leq d\\ {\left(x-d\right)}^{2}\;\mathrm{for}\;x>d \end{array}\right.$$

If the predicted distance (x) is within the threshold (d), the penalty is 0. If it exceeds, the penalty is the squared error between the threshold (d) and the predicted distance (x). The final HDX penalty for each docking model is the sum of individual HDX peptide penalties.

Each model’s penalty was normalized by dividing the raw value by the maximum penalty observed among all models for the benchmark complex. This normalization, expressed as a percentage, enabled comparison of HDX penalties across different restraint settings.

In all test cases, HDX restraints showed desirable distinguishing power, where penalties increased progressively as model interface RMSD increased (Fig. S2). However, for FluA-20, H5.28, H5.3, and H5.31 complexes, more stringent restraints, i.e., reducing the threshold distance to 8 Å and 5 Å, introduced slight penalties for near-native models (iRMSD from 0–4 Å). Each of these complexes contains an allosteric peptide, as illustrated in Fig. S3. The inappropriate penalization of near-native models in these cases suggested that 8 Å and 5 Å restraints were too stringent for complexes with allosteric peptides, resulting in high false-positive penalties.

At a threshold distance of 10 Å, near-native models received no penalty, and the restraints maintained their effectiveness in distinguishing model quality across different minimum residue settings (Fig. S2). Therefore, we selected the most relaxed HDX restraint, which requires at least one residue (Cα) in each HDX peptide to be within 10 Å of another residue (Cα) in the Ab CDRs. This setting optimized HDX data utility while mitigating potential misguidance from allosteric peptides.

### 4.5. Rescoring of docking models with combined HDX and Rosetta interface score

To assess the agreement between Rosetta docking models with experimental HDX data, HDX score was computed for the final docking models using the respective HDX restraints. For each docking experiment, the raw HDX score was normalized to the Rosetta interface score (dG_separated) using a different normalizing weight, which was adjusted such that the ranges of the HDX and Rosetta interface score were approximately equal. The normalized HDX score for each docking experiment was calculated as

(2) $$normalized\_HDX\_score=\frac{{Rosetta\_interface\_score}^{high}\;-{\;Rosetta\_interface\_score}^{low}}{{HDX\_score}^{high}\;-{\;HDX\_score}^{low}}\times HDX\_score$$

where ${Rosetta\_interface\_score}^{high}$ and ${Rosetta\_interface\_score}^{low}$ are the average of the highest and lowest 10 % of the values of the Rosetta interface score, and ${HDX\_score}^{high}$ and ${HDX\_score}^{low}$ are the average of the highest and lowest 10 % of the values of the respective HDX score.

The normalized HDX score was then weighted by a weight value (which was ultimately optimized to 4.5), and added to the Rosetta interface score:

(3) combined_score=(4.5×normalized_HDX_score)+Rosetta_interface_score

To test the effectiveness of the new combined score for model selection, 10,000 Rosetta models, generated with and without HDX restraints, were rescored using the combined score. We explored a range of potential weight values from 0.5 to 15, in increments of 0.5. At each weight, we assessed improvements by examining the enrichment values (max value = 10) and interface RMSDs of the top 10 models selected by the new score (Fig. S5).

For models built without HDX restraints, there was scoring improvement (i.e., a decrease in top 10 scoring model average iRMSD and an increase in enrichment value) at nearly every weight tested (Fig. S5A). As the weight increased from 0.5 to 4.5, the degree of improvement initially rose, stabilizing around 4.5, and remained fairly constant from 4.5 to 15.0. The same trend applied to models generated with HDX restraints, though not all complexes improved—those that did not generally remained unchanged (Fig. S5B). An exception was H5.3, where the scoring power declined when the weight exceeded 5.5, due to inappropriate penalization of good models. This aligned with our previous discussions on the limitations of HDX discrimination power, particularly when allosteric peptides are included and overly weighted in docking simulations. To mitigate the misleading influence of allosteric peptides in HDX-based scoring, particularly when HDX was previously used for sampling, a balanced weight is critical. A weight of 4.5 was selected as optimal for rescoring and was used in RosettaHDX. It was the lowest weight that consistently yielded improvements in top scoring models’ iRMSDs and enrichment values across most complexes, without worsening results in either simulation setting (with or without HDX restraints) (Fig. 3A and S3C).

### 4.6. Rescoring of docking models with HADDOCK, ZDOCK, and AF2Rank

Docking models generated with HDX restraints using AF individual subunit structures were rescored with HADDOCK (de Vries et al., 2010; Dominguez et al., 2003a; van Zundert et al., 2016), ZDOCK (Pierce et al., 2011), and AF2Rank (Roney and Ovchinnikov, 2022). HADDOCK scores were obtained using the default scoring script for protein–protein complexes in HADDOCK3 (Dominguez et al., 2003b), available on GitHub (https://github.com/haddocking/haddock3). ZRANK scores were computed using the ZRANK2 executable, part of ZDOCK 3.0.2 (Pierce and Weng, 2008; Pierce et al., 2011), downloaded from https://zdock.wenglab.org/software/download/. AF confidence scores for protein complexes were calculated using AF2Rank with default parameters (Roney and Ovchinnikov, 2022), available on GitHub (https://github.com/jproney/AF2Rank).

### 4.7. Performance metrics for docking

To assess the performance of HDX data in Ab-Ag docking, several evaluation metrics were used. The prediction accuracy (interface RMSD) was quantified using the RMSD of backbone Cα atoms in the interface after superposition. The interface was defined as all residues having at least one atom within 10 Å of an atom on the other protein binding partner. The results of the docking benchmark were also evaluated by near-native model count. To be counted as near-native, the docking models must meet the standard criteria for a CAPRI acceptable, medium-quality, or high-quality models based on the fraction of correctly predicted native contacts, ligand RMSD, and interface RMSD (Méndez et al., 2003).

The effect of HDX score on model scoring was assessed using the average interface RMSD of top 10 scoring models and the enrichment value. Enrichment metric is defined as (TP/(TP + FP) x (P + N)/P). Therefore, models were sorted according to either their Rosetta score or their combined Rosetta and HDX score. Models that fell within the top 10 % by score were counted as “positive” (P) and the remaining models were counted as “negative” (N). The positives were then sorted by interface RMSD, and those models that fell within the top 10 % by interface RMSD were labeled “true positives” (TP); all other models were considered “false positives” (FP). Since the (P + N)/P ratio is set to 10, the maximum possible enrichment is also limited to a value of 10.

Calculation of the mean (± S.D.) interface RMSD of the best 1 % models by iRMSD and the ten top-scoring models listed in Table S2 and S4 and displayed in Figs. S4, S8, and S10 was carried out with Numpy for Python3.7. Statistical significance of the difference between the iRMSD-based cumulative distributions of models generated with and without HDX restraints, was evaluated using a two-sample Kolmogorov-Smirnov test. The test, along with the Kolmogorov-Smirnov statistic value and p-value, was conducted using SciPy in Python 3.10. The Kolmogorov-Smirnov statistic value ranges from 0 to 1, with larger values indicating greater discrepancies between the iRMSD-based cumulative distributions of models generated with and without HDX restraints. Statistical significance of the difference in model enrichment, iRMSD, number of near-native models for calculations with and without HDX restraints was evaluated by a two-tailed Wilcoxon signed-rank test and carried out with Scipy for Python3.7. Significance values (* p < 0.05, ** p < 0.01, *** p < 0.001) are listed in Table S2 and displayed in Figs. 3, S4, and S9.

To compare docking performance between RosettaHDX and AF-Multimer, docking runs with at least one near-native model among the top 10 scoring models are categorized as “successful”.

### 4.8. AF modeling of Ab-Ag complex and unbound Ab and Ag models

AF predictions were performed in this study using AF2 (v.2.3). As a comparison to our RosettaHDX method, we generated full Ab-Ag structures for each benchmark complex using AF in Multimer setting (AF-Multimer).

To generate unbound Ab and Ag structures, AF-Multimer was employed when the input was a heavy-light chain Ab or a multimeric Ag. Specifically, in the case of FI6V3 complexes, the Ag is a full-length HA1 and HA2 dimer; thus, AF-Multimer was used. Alternatively, the Monomer setting was used when the Ag was a single chain. All AF2 protocols and calculations were performed using default settings as described in Abramson et al., 2024 and Jumper et al., 2021, producing 5 output models for the Monomer setting and 25 for the Multimer setting (Abramson et al., 2024; Jumper et al., 2021).

To resolve potentially unfavorable geometries or clashes in AF models, the Rosetta FastRelax protocol (Tyka et al., 2011) was applied to AF predicted structures prior to scoring of the models in Rosetta using interface analysis protocols. Post-relax AF Ab-Ag complex structures were used as inputs to obtain Rosetta InterfaceAnalyzer protocol scores. The “InterfaceAnalyzer” executable in Rosetta v. 3.13, with default parameters computes and outputs interface energetic scores using the Rosetta REF15 function, along with REF15 component terms and other interface structure metrics (Schoeder et al., 2021).

The top five unbound Ab and Ag models ranked by AF confidence scores (pLDDT for AF monomer and ipTM + pTM for AF-Multimer) were selected to proceed with docking. These five post-relax Ab models were paired with five corresponding Ag models to create an initial docking ensemble of 25 starting conformations. The CDRs and the framework regions of Abs were identified by Chothia numbering (Honegger and Plückthun, 2001), assigned using abYsis web-based Ab research system (Swindells et al., 2017). Backbone RMSDs of CDR loops between the AF Ab model and the native structure were calculated after superposing the framework regions of the two structures.

### 4.9. Predictive metric for allosteric peptides

To predict the presence of an allosteric peptide in the HDX dataset from docking experiment results, we developed a predictive metric independent of the native structure. The top 10 scoring models were selected based on the combined Rosetta interface and HDX score. The normalized, weighted HDX scores for these models were then averaged. An average weighted HDX score greater than 0.75 indicated the presence of an allosteric peptide in the HDX dataset.

To identify the specific allosteric peptide within the HDX dataset, we systematically excluded each peptide and recalculated the weighted HDX score for each docking model. This updated HDX score was then combined with the Rosetta interface score to select a new set of top 10 scoring models. The average weighted HDX score for this updated set was evaluated, and the peptide whose exclusion resulted in the lowest average HDX score was predicted to be the allosteric peptide. If the new average HDX score remains above 0.75, the process is repeated to identify the next allosteric peptide. Redocking should be performed with the updated HDX restraints set after each allosteric peptide identification to enhance the quality of the generated docking ensemble.

## Supplementary Material

Appendix A supplementary material

## Figures and Tables

**Table 1:** CAPRI criteria for docking model quality.

Model quality	fnat	L-RMSD	or iRMSD

High	≥ 0.5	≤ 1.0	or ≤ 1.0
Medium	≥ 0.3	1.0 < x ≤ 5.0	or 1.0 < x ≤ 2.0
Acceptable	≥ 0.1	5.0 < x ≤ 10.0	or 2.0 < x ≤ 4.0
Incorrect	< 0.1		

## Data Availability

Data will be made available on request.

## Abbreviations:

HDX-MS: hydrogen-deuterium exchange mass spectrometry
Ab-Ag: antibody-antigen
