# Supplementary material for: RosettaHDX: Predicting antibody-antigen interaction from hydrogen-deuterium exchange mass spectrometry data
Source: J Struct Biol. Author manuscript; Available in PMC 2025 Apr 21. (PMC12010952; doi:10.1016/j.jsb.2025.108166)
Supplement: Appendix A supplementary material [file NIHMS2069322-supplement-Appendix_A_supplementary_material.zip › 1-s2.0-S1047847725000012-mmc1.docx]

**SUPPLEMENTARY INFORMATION**

**RosettaHDX: Predicting Antibody-Antigen Interaction from Hydrogen-Deuterium Exchange Mass Spectrometry Data**

**Minh H. Tran^1,2,*^, Cristina E. Martina^2,3^ , Rocco Moretti^2,3^, Marcus Nagel^4^, Kevin L. Schey^4,*^, Jens Meiler^2,3,5,6,7*^**

^1^Chemical and Physical Biology Program, Vanderbilt University, Nashville, Tennessee, USA

^2^Center of Structural Biology, Vanderbilt University, Nashville, Tennessee, USA

^3^Department of Chemistry, Vanderbilt University, Nashville, Tennessee, USA

^4^Mass Spectrometry Research Center, Department of Biochemistry, Vanderbilt University, Nashville, Tennessee, USA

^5^Institute for Drug Discovery, Institute for Computer Science, Wilhelm Ostwald Institute for Physical and Theoretical Chemistry, University Leipzig, Leipzig, Germany

^6^Center for Scalable Data Analytics and Artificial Intelligence ScaDS.AI and School of Embedded Composite Artificial Intelligence SECAI, Dresden/Leipzig, Germany

^7^Department of Pharmacology, Institute of Chemical Biology, Center for Applied Artificial Intelligence in Protein Dynamics, Vanderbilt University, Nashville, Tennessee, USA

* Corresponding author(s). E-mail(s): [minh.h.tran@vanderbilt.edu](mailto:minh.h.tran@vanderbilt.edu) (lead contact); [k.schey@vanderbilt.edu](mailto:k.schey@vanderbilt.edu); [jens@meilerlab.org](mailto:jens@meilerlab.org)

**TABLE OF CONTENTS**

1. Supplementary Results
   1. HDX restraints improved sampling when docking with AF models as input
   2. HDX combined score improved model selection when docking with AF models as input
   3. Rationale for developing the predictive metric for allosteric peptides
   4. Performance of the predictive metric for HDX allosteric peptides when docking with co-crystal structures as input
2. Additional Figures
   1. S1 HDX-MS analysis of different Abs binding footprint to HA monomeric head
   2. S2 Testing different HDX restraint parameters in distinguishing model quality
   3. S3 Statistical analysis of HDX restraints enhancing sampling and scoring when docking with co-crystal structures as input
   4. S4 Panel of Ab-Ag benchmark complexes and their corresponding HDX peptides
   5. S5 Optimization of the combined score of HDX and Rosetta interface score for model selection
   6. S6 Top 10 models selected using Rosetta interface score versus the combined HDX and Rosetta interface score when docking with co-crystal structures as input
   7. S7 Sampling with and without HDX restraints when docking with AF models as input
   8. S8 Statistical analysis of HDX restraints enhancing sampling when docking with AF models as input
   9. S9 Top 10 models selected using Rosetta interface score versus the combined HDX and Rosetta interface score when docking with AF models as input.
   10. S10 Statistical analysis of HDX restraints enhancing scoring when docking with AF models as input
   11. S11 Rosetta interface score versus weighted HDX score when docking with HDX restraints and with co-crystal structures as input
   12. S12 Average weighted HDX score versus iRMSD of the top 10 scoring models generated by RosettaHDX starting from co-crystal structures
   13. S13 HADDOCK score versus iRMSD for docking ensemble generated with HDX restraints starting from AF models
   14. S14 ZRANK score (from ZDOCK) versus iRMSD for docking ensemble generated with HDX restraints starting from AF models
   15. S15 AF confidence score (from AF2Rank) versus iRMSD for docking ensemble generated with HDX restraints starting from AF models
3. Additional Tables
   1. S1 Summary of Ab-Ag benchmark complexes and corresponding HDX experimental results used in docking
   2. S2 Summary of docking results starting from co-crystal structures with and without HDX restraints for sampling and scoring
   3. S3 Number of near-native models (CAPRI high, medium, and acceptable quality) generated with and without HDX restraints for each of the nine benchmark complexes
   4. S4 Summary of docking results starting from AF models with and without HDX restraints for sampling and scoring
   5. S5 Average weighted HDX score of the top 10 scoring model generated with RosettaHDX
4. Protocol Capture - RosettaHDX Tutorial

**Supplementary Results**

**HDX restraints improved sampling when docking with AF models as input**

When Ab and Ag models were used as docking inputs, our HDX restraints continued to effectively guide sampling, resulting in a larger number of higher-quality models across all benchmark complexes.

Table S4 summarizes the average iRMSD of the top 1% of models ranked by their interface-RMSD relative to the native structure. In all nine cases, the inclusion of HDX restraints significantly improved the average iRMSD of the top 1% of models, reducing it from 4.6 Å to 3.4 Å (Figure S8 and Table S4). This enhancement in model sampling is further illustrated in the interface score versus iRMSD and density plots shown in Figure S7. Significant improvements in the iRMSD distribution of the AF model docking ensembles generated with HDX data were observed as a leftward shift in the density curves. These improvements were statistically significant, as determined by the two-sample Kolmogorov-Smirnov test (p < 0.001) for all complexes. The Kolmogorov-Smirnov statistic values ranged from 0.14 (H7.200) to 0.64 (FI6V3_3ztn) across the nine complexes. Additionally, HDX restraints narrowed the iRMSD sampling range for benchmark complexes. For example, in the FI6V3 complexes, the iRMSD range for generated models was reduced from 1.5–60.7 Å to 1.5–37.0 Å with HDX restraints. Similar improvements were seen in other complexes, with an average sampling range decrease of ~5 Å. When docking with model inputs, sampling improvements with HDX restraints were still observed in cases involving allosteric peptides (H5.28, H5.3, H5.31, and FluA-20).

Not only did HDX restraints lower the overall iRMSD of the generated models, but they also increased the generation of near-native models for most complexes, resulting in approximately 3.6 times more near-native models overall (Table S3). This enhanced sampling of near-native models was also reflected in the increased population of models within the iRMSD range of 0–5 Å, as shown in the density plot in Figure S7, with increases ranging from 1.5-fold (H7.200) to 6.3-fold (H5.3 and FI6V3_3ztn). Furthermore, for the H5.3 and H7-200 complexes, HDX restraints enabled the generation of high-quality models (iRMSD ≤ 1 Å) that were not achievable with RosettaDock alone.

Due to structural deviations in the docking inputs, the number of near-native decoys was lower when docking with Ab and Ag models, as expected (the average iRMSD of top 1% iRMSD models was 3.4 Å compared to 2.6 Å with co-crystal structure derived input).

**HDX combined score improved model selection when docking with AF models as input**

When applying HDX combined score for model selection, the average enrichment across the benchmark set significantly increased from 1.7 to 3.2, nearly doubling for models generated without HDX restraints, and from 1.4 to 1.8 for models generated with HDX restraints (Figure S9A and Table S4). The top ten models selected for each benchmark complex before and after HDX rescoring are shown in Figure S9B, with their average iRMSD values listed in Table S4. For models generated without HDX restraints, HDX rescoring reduced the average iRMSD across all benchmark complexes, improving from 14.1 Å to 10.6 Å. For models generated with HDX restraints, 6 out of 9 complexes showed a reduction in average iRMSD, with an overall improvement from 10.9 Å to 10.3 Å. In docking without HDX restraints, Rosetta successfully predicted one benchmark complex (H5.3), and using the HDX score to select models increased the number of near-native models in the top ten for this complex. When docking with HDX restraints, Rosetta successfully predicted three benchmark complexes, and using the HDX score to select models increased the number of near-native models in the top ten for 2 out of the 3 complexes. In summary, incorporating the HDX score into Rosetta’s energy function significantly improved the quality of models selected from the structure ensemble, even when docking with model inputs.

**Rationale for developing the predictive metric for allosteric peptides**

We examined the Rosetta binding energy and weighted HDX score (summed from all HDX peptides in each set) for 10,000 docked models using RosettaHDX, starting from crystal structures (Figure S11). An HDX score of 0 indicates no HDX violations. As shown in Figure S11, all near-native models (colored dots) aligned vertically at an HDX score of 0 for the five complexes without allosteric peptides (upper row). In contrast, near-native models for complexes with allosteric peptides (lower row) occupied a broad distribution of higher HDX scores. This was reasonable because, while native-like models conformed to restraints from true interacting peptides, they violated those imposed by allosteric peptides, which were not part of the native binding interface. Consequently, we speculated that the weighted HDX score by itself may serve as a predictive metric for identifying cases where allosteric peptides are present.

It is important to note that the HDX score alone cannot distinguish non-native poses from native ones, as many decoy models in Figure S11 exhibited a favorable HDX score of 0 despite having high iRMSD values. This was not unexpected, as the sparse information obtained from HDX was not comprehensive of the entire complex structure. Instead, the HDX score primarily reflected violations of HDX data in the models.

**Performance of the predictive metric for HDX allosteric peptides when docking with co-crystal structures as input**

For RosettaHDX docking simulations starting from crystal structures, our approach successfully identified the allosteric peptides for H5.3 and H5.31. Excluding their allosteric peptides from the HDX score resulted in the lowest HDX score for the newly selected top 10 scoring models (Table S4). In docking runs starting from crystal structures, all top 10 models selected after accurately predicting the allosteric peptide and excluding it from HDX scoring had the allosteric peptide positioned more than 10 Å away from the binding CDRs. In the case of H5.3, omitting the allosteric peptide did not significantly improve iRMSD, as the original selection already included 10 high-quality models in the top 10, which remained unchanged after rescoring. For H5.31, the original combined HDX score selected 9 near-native models in the top 10. However, when rescoring with the allosteric peptide excluded, one incorrect model with a low Rosetta interface score that also violated the allosteric peptide restraints was now optimally scored and included in the top 10. This reduced the number of near-native models from 9 to 8, resulting in a slight increase in the average iRMSD.

**Additional Figures**
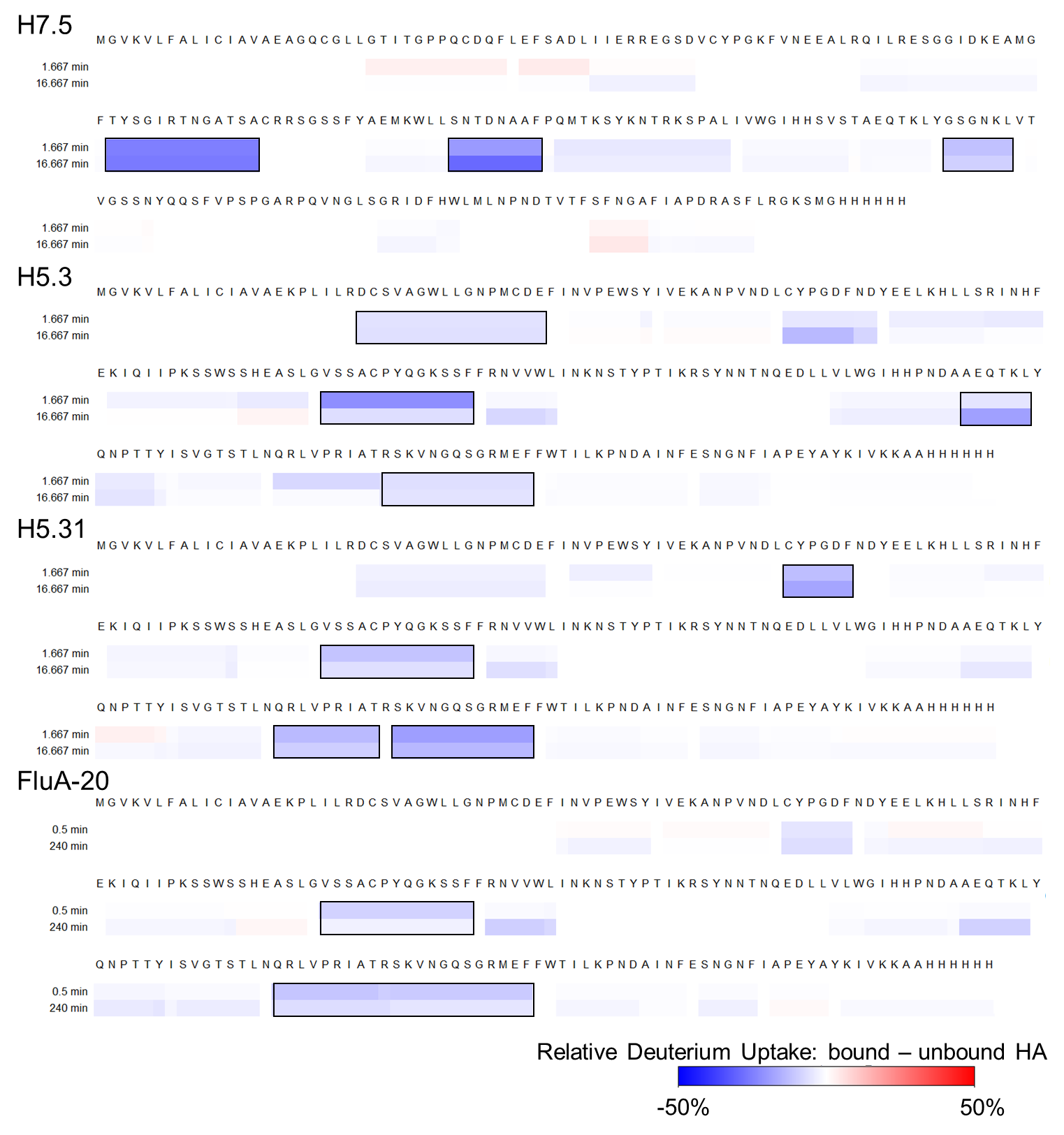


**Figure S1. HDX-MS analysis of different Abs binding footprint to HA monomeric head.** HDX exchange heat map in linear sequence showing the difference in relative deuterium-uptake of HA in complex with H7.5, H5.3, H5.31, and FluA-20, compared to unbound HA. The sequences of corresponding HA strain for each complex in the HDX experiment are shown. The relative fractional uptake difference (%) is colored from blue to red. Potential interacting peptides for HA in each complex are highlighted in boxes, defined by at least one time point with a relative deuterium uptake difference ≥0.5 Da ± 3.0 standard deviations when comparing the bound versus unbound HA, with a cumulative difference of >1.1 Da over the entire exposure time.


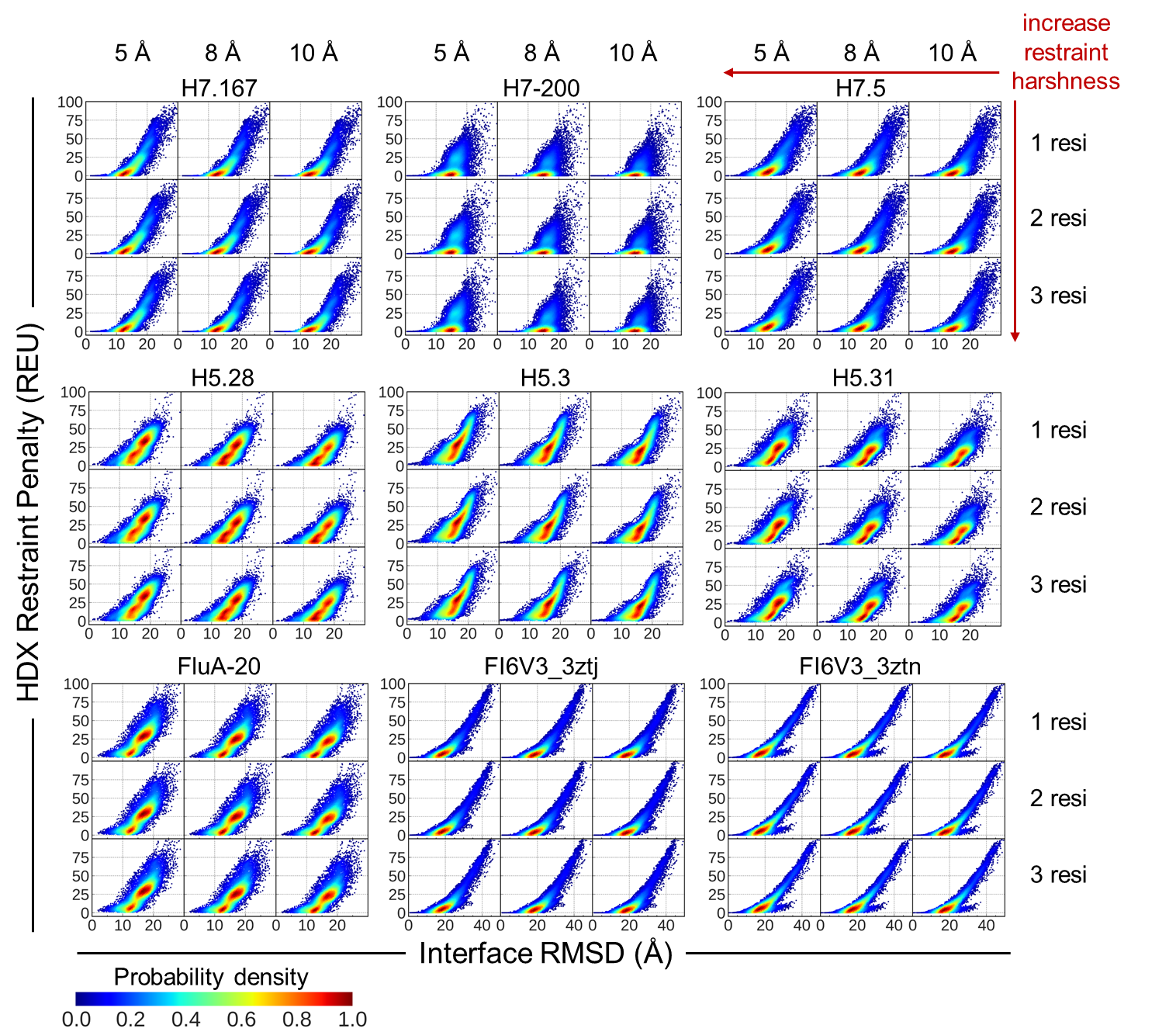


**Figure S2: Testing different HDX restraint parameters in distinguishing model quality.** HDX restraint penalty vs. iRMSD plots for 10,000 docked models generated without HDX guidance, across nine benchmark complexes. For each benchmark complex, nine different settings of HDX restraints were tested. The most lenient setting required the Cα of at least one amino acid (excluding Proline) in each HDX-interacting peptide on the Ag to be within 10 Å of any Cα in the Ab's complementarity-determining regions (CDRs). More stringent settings increased the required number of interface residues in each HDX peptide from 1 to 2 to 3 and reduced the Cα–Cα threshold distance from 10 Å to 8 Å to 5 Å. For each parameter, the HDX penalty was normalized to the maximum observed among the 10,000 models, shown as a percentage within the set. The increase in HDX penalties relative to the decrease in model quality (iRMSD) indicates how effectively each restraint setting distinguishes model quality. This distinguishing power was evaluated across different restraint settings. Models in each plot are color-coded based on their density distribution.

**
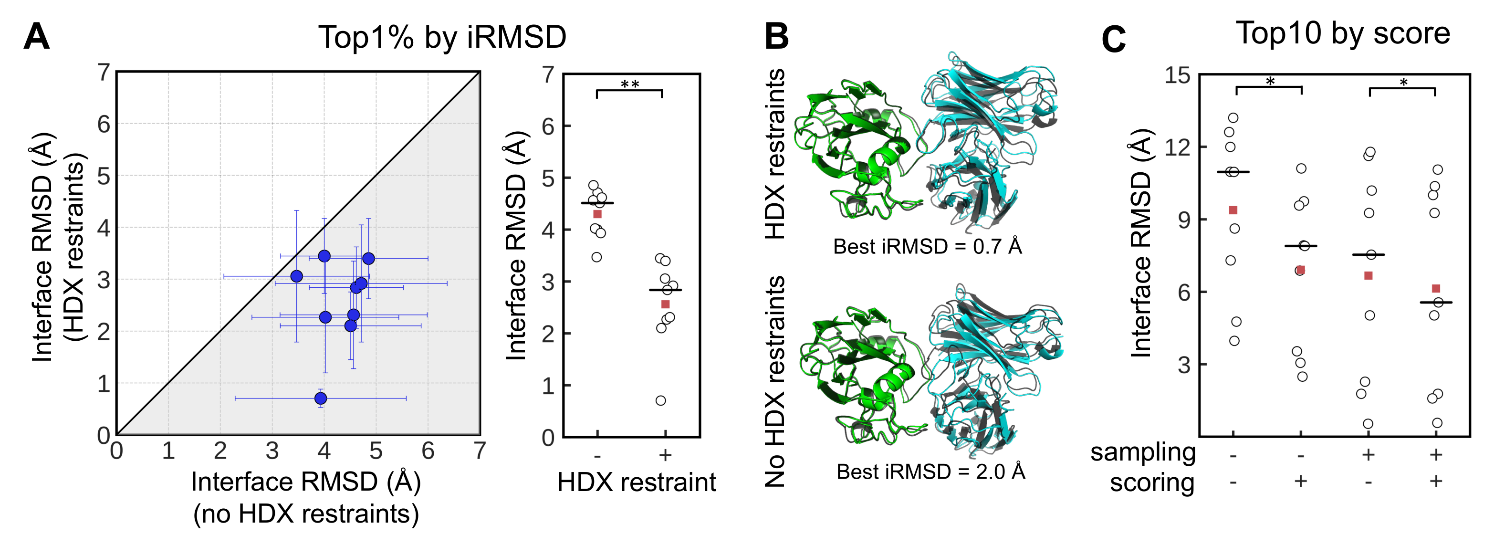

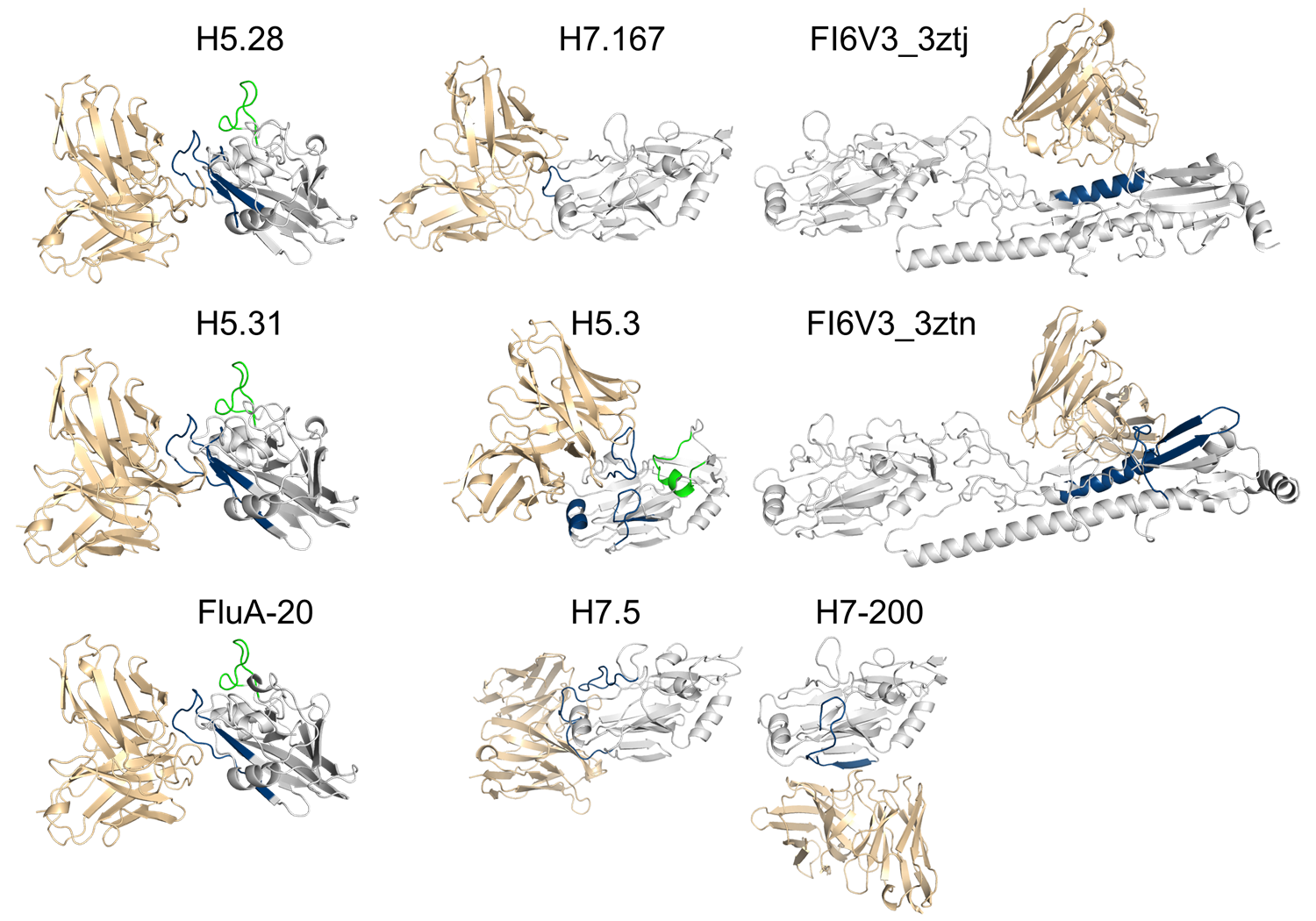
Figure S3: Statistical analysis of HDX restraints enhancing sampling and scoring when docking with co-crystal structures as input.** (A) Comparison of the average interface iRMSD (±SD) of the top 1% of models ranked by iRMSD between docking models predicted without HDX data and with HDX data. Gray area indicates an iRMSD improvement (left plot). Statistical analysis for this comparison is shown (right plot). (B) The best iRMSD docking model generated from crystal structure for FluA-20, with and without HDX restraints applied during sampling. (C) Comparison of the average iRMSD of the top 10 models by score using Rosetta interface score alone versus the combined HDX and Rosetta interface score. This comparison was performed for models generated without HDX restraints in sampling and for models generated with HDX restraints in sampling. The iRMSD distribution median (**―**) and average (🟥) are marked. Statistical significance was assessed using a two-tailed Wilcoxon signed rank test (* p < 0.05, ** p < 0.01).

**Figure S4: Panel of Ab-Ag benchmark complexes and their corresponding HDX peptides.** The experimentally determined structures of the nine Ab-Ag benchmark complexes are shown. Abs are colored yellow, HAs are colored grey, with HDX true interacting peptides in dark blue, and allosteric peptides in green.


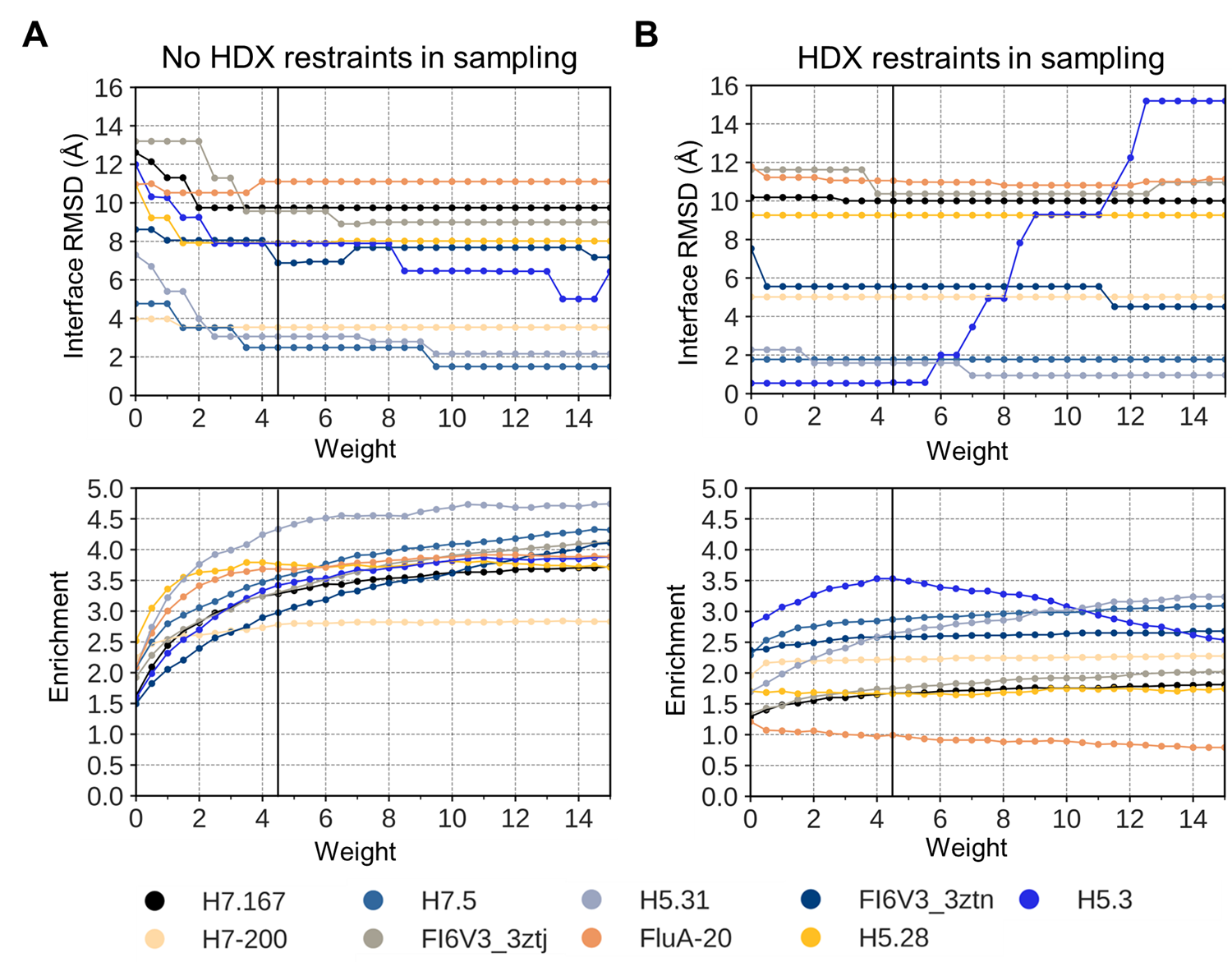


**Figure S5. Optimization of the combined score of HDX and Rosetta interface score for model selection.** Plots of average iRMSD of top 10 scoring models and enrichment evaluated before (weight=0) and after rescoring with the combined score at various weights for HDX score. (A) Results for models generated without HDX restraints in sampling. (B) Results for models generated with HDX restraints in sampling. The vertical line at a weight of 4.5 marks the optimal and lowest weight that achieves the best model selection accuracy (low iRMSD and high enrichment).


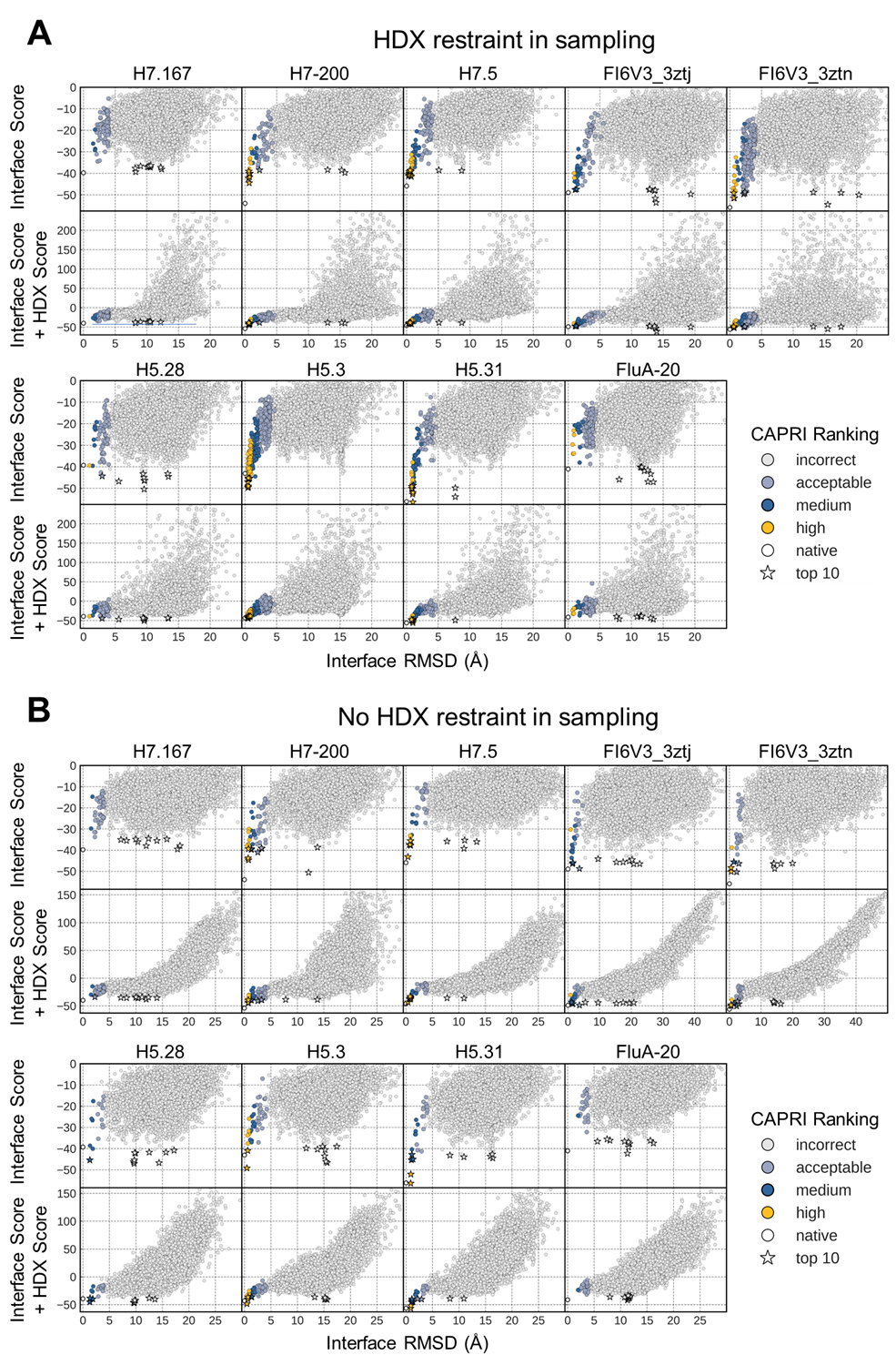
**Figure S6. Top 10 models selected using Rosetta interface score versus the combined HDX and Rosetta interface score when docking with co-crystal structures as input.** Score vs. iRSMD for 10,000 models generated (A) with HDX restraints and (B) without HDX restraints during sampling. The colors indicate model accuracy based on CAPRI criteria (Janin et al., 2003), as detailed in the legend: high (yellow), medium (dark blue), acceptable (light blue), and incorrect (light gray). For each benchmark complex, the top 10 scoring models selected by Rosetta interface score and by the combined HDX score are marked with star markers and are colored according to the CAPRI criteria. The white circle represents the reference energy of the relaxed, bound crystal structure.


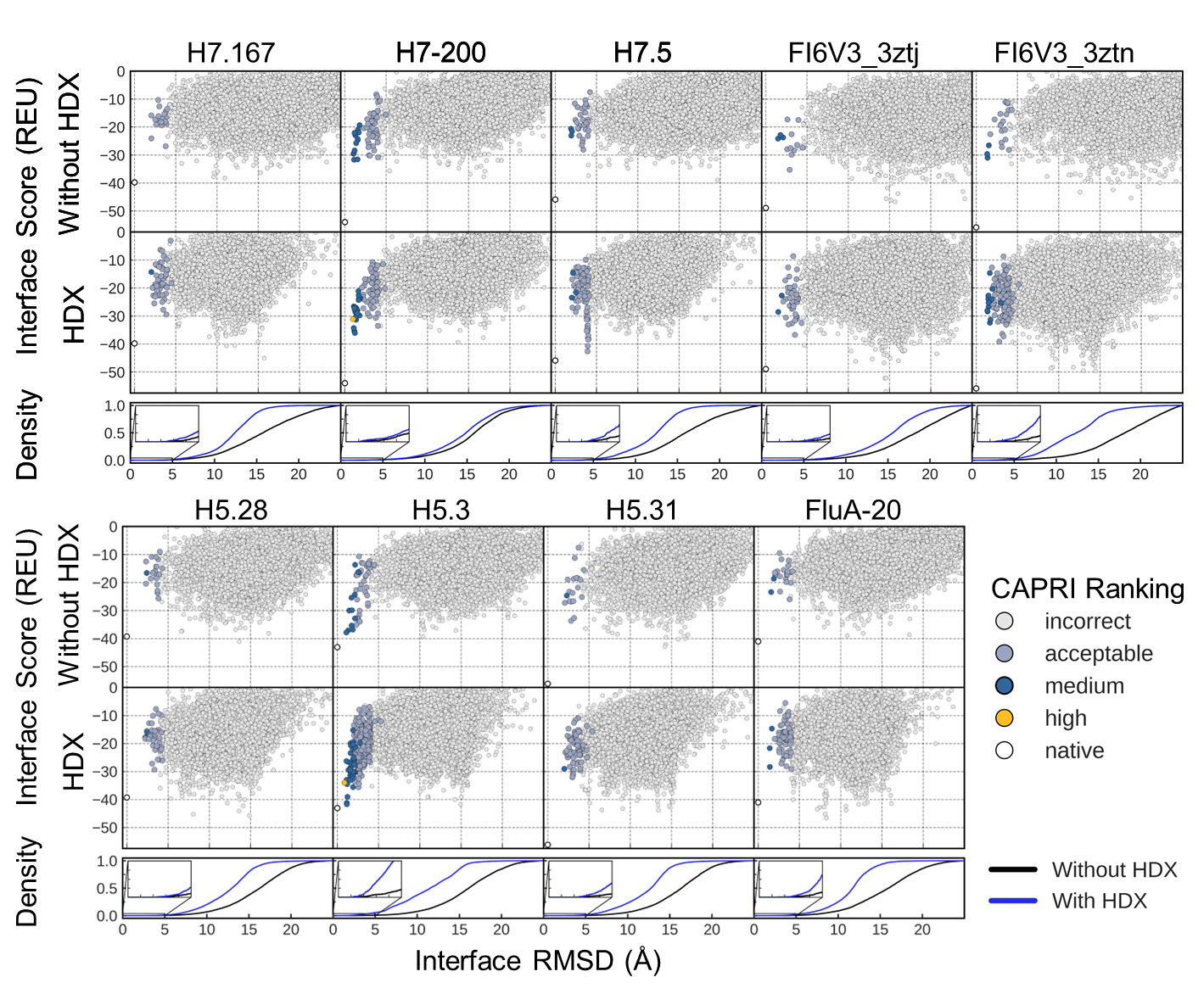


**Figure S7. Sampling with and without HDX restraints when docking with AF models as input.** The figure displays Rosetta binding interface score versus interface RMSD plots, and cumulative fraction of models versus interface RMSD plots for 10,000 models generated from AF models with and without HDX distance restraints. Model accuracy is color-coded based on CAPRI criteria (Janin et al., 2003), as detailed in the legend: high (yellow), medium (dark blue), acceptable (light blue), and incorrect (light gray). The white circle marks the reference energy of the relaxed, bound crystal structure. The density plots display the cumulative fraction of models within the displayed iRMSD range of 0 to 25 Å, comparing docking results without HDX distance restraints (black) to those with HDX restraints (blue).


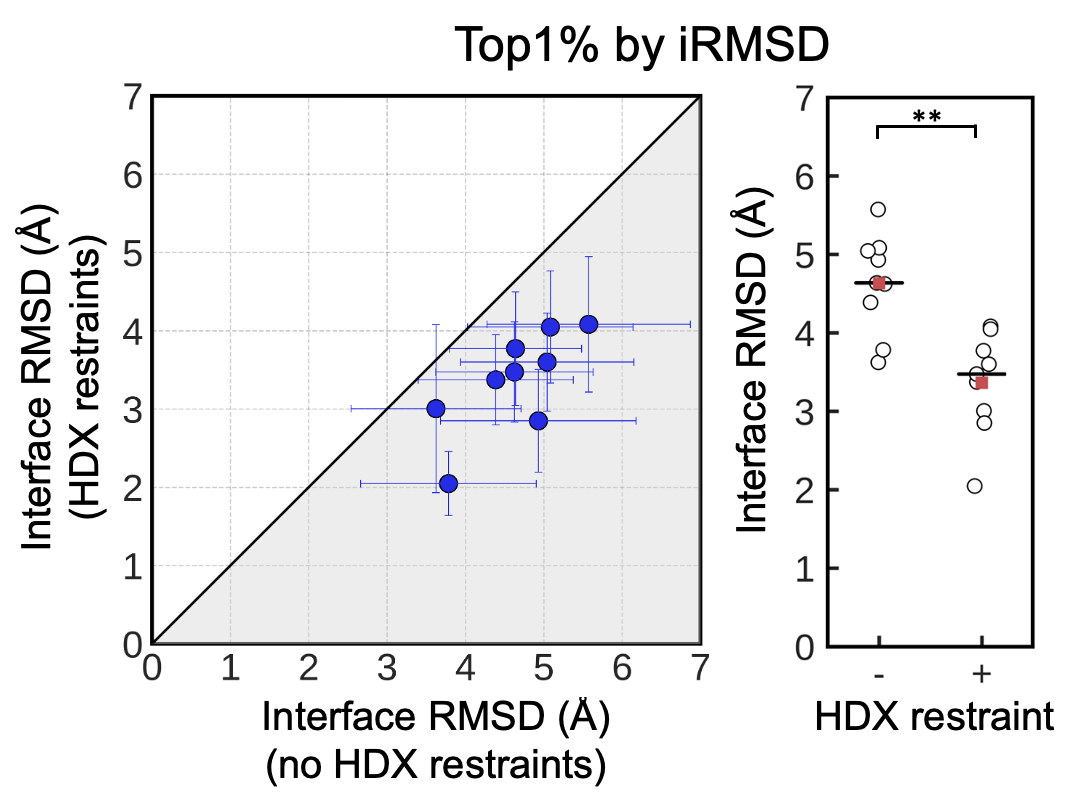


**Figure S8. Statistical analysis of HDX restraints enhancing sampling when docking with AF models as input.** Comparison of the average interface iRMSD (±SD) of the top 1% of models ranked by iRMSD between docking models predicted without HDX data and with HDX data. Gray area indicates an iRMSD improvement (left plot). Statistical analysis for this comparison is shown (right plot).The iRMSD distribution median (**―**) and average (🟥) are marked. Statistical significance was assessed using a two-tailed Wilcoxon signed rank test (* p < 0.05, ** p < 0.01).


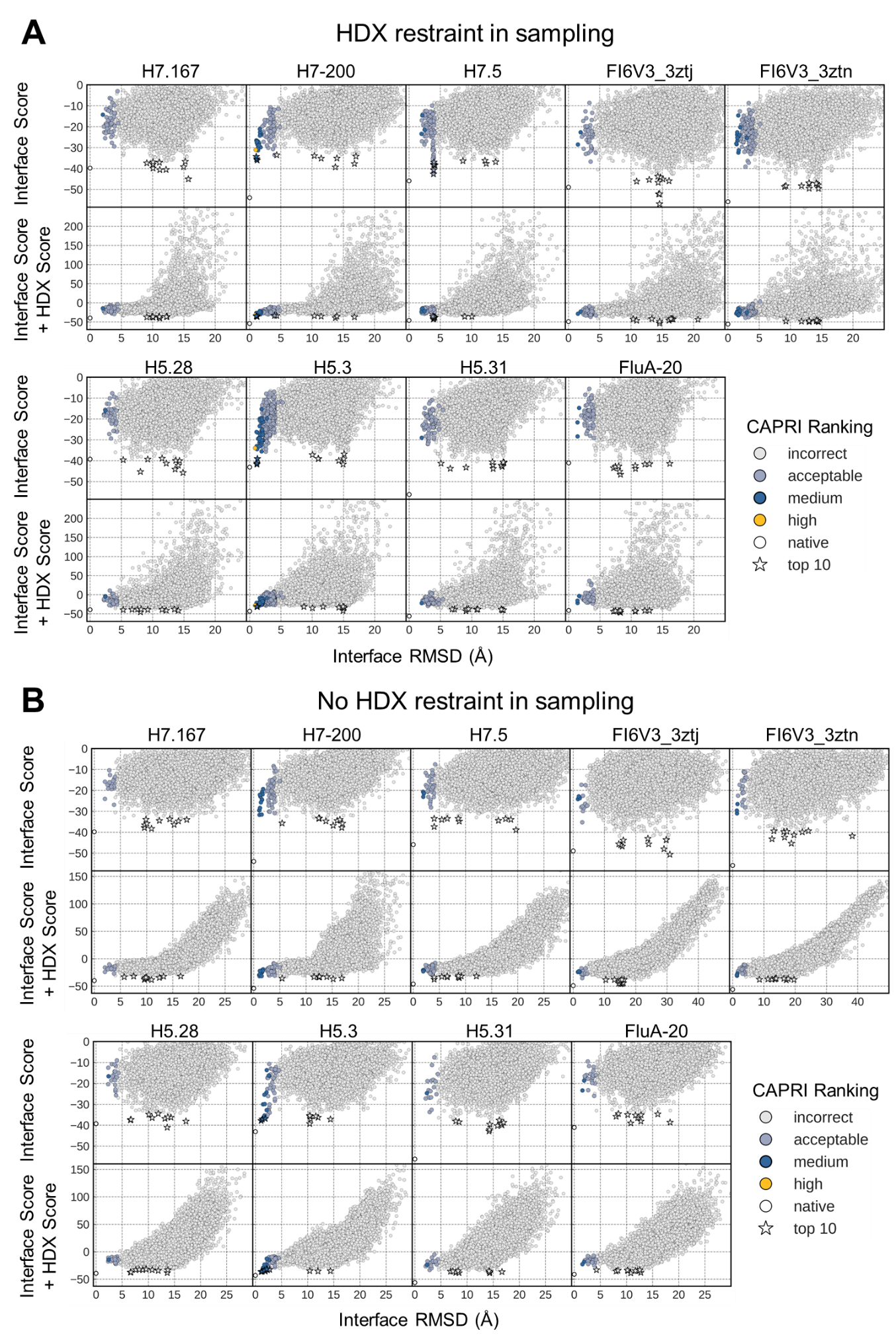


**Figure S9. Top 10 models selected using Rosetta interface score versus the combined HDX and Rosetta interface score when docking with AF models as input.** Score vs. iRSMD for 10,000 models generated (A) with HDX restraints and (B) without HDX restraints during sampling. The colors indicate model accuracy based on CAPRI criteria (Janin et al., 2003), as detailed in the legend: high (yellow), medium (dark blue), acceptable (light blue), and incorrect (light gray). For each benchmark complex, the top 10 scoring models selected by Rosetta interface score and by the combined HDX score are marked with star markers and colored according to the CAPRI criteria. The white circle represents the reference energy of the relaxed, bound crystal structure.


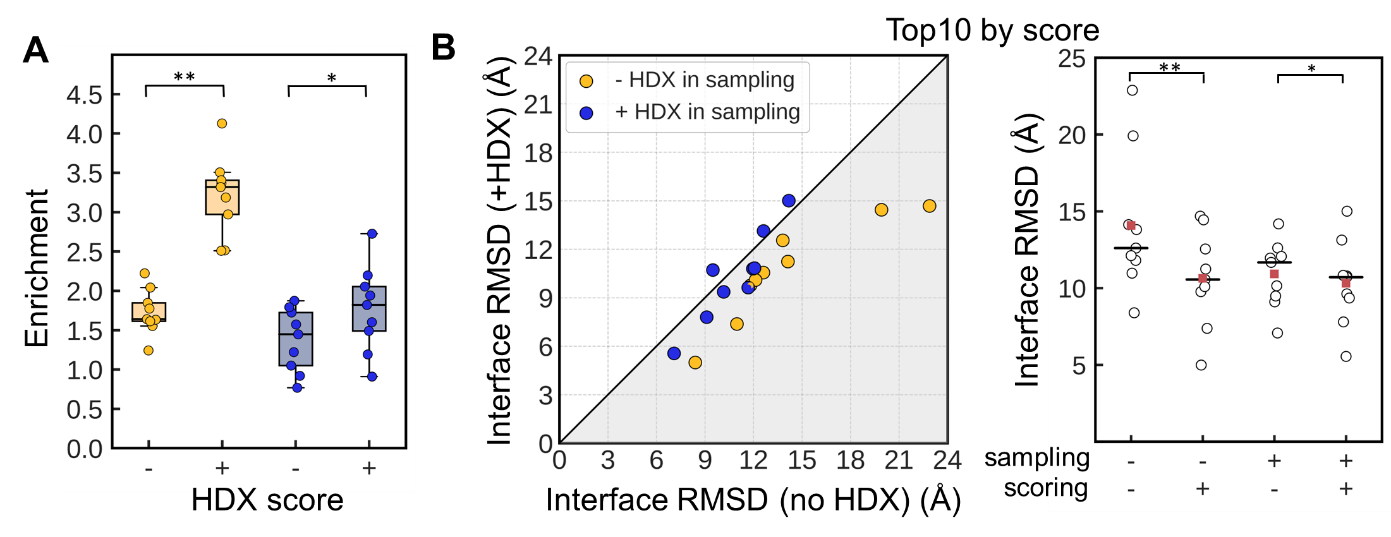


**Figure S10. Statistical analysis of HDX restraints enhancing scoring when docking with AF models as input.** Docking ensembles predicted with and without HDX data during sampling are shown in blue and yellow, respectively. (A) Model enrichment was calculated when models were scored using Rosetta interface score alone and with the addition of the HDX score. The box-and-whisker plot displays whiskers extending to 1.5 times the interquartile range of the lower and upper quartiles. (B) Comparison of the average interface RMSD among the top ten scoring models when scored with Rosetta interface score alone and with the addition of the HDX score. Gray area indicates iRMSD improvement of the top ten scoring models with the addition of the HDX score (left plot). Statistical analysis for this comparison is shown (right plot). This comparison was performed for models generated without HDX restraints in sampling and for models generated with HDX restraints in sampling. The iRMSD distribution median (**―**) and average (🟥) are marked. Statistical comparisons were assessed using a two-tailed Wilcoxon signed-rank test (n=9, *p < 0.05, **p < 0.01)


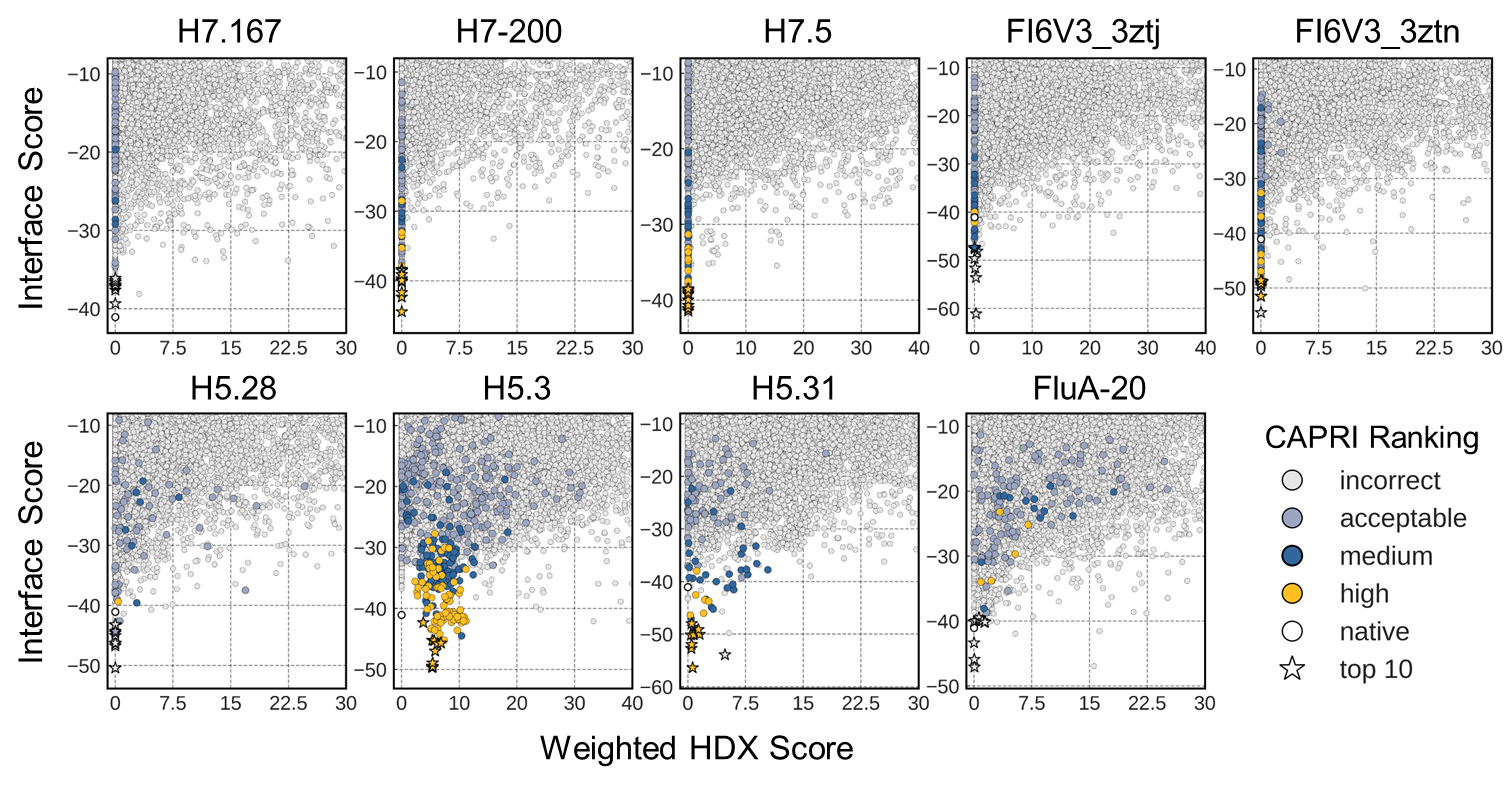
**Figure S11. Rosetta interface score versus weighted HDX score when docking with HDX restraints and with co-crystal structures as input.** A total of 10,000 models were generated with HDX restraints applied during sampling, using crystal structures as docking input. The colors indicate model accuracy based on CAPRI criteria (Janin et al., 2003), as detailed in the legend: high (yellow), medium (dark blue), acceptable (light blue), and incorrect (light gray). For each benchmark complex, the top 10 scoring models selected by the combined HDX and Rosetta interface score are marked with star markers and colored according to the CAPRI criteria. The white circle represents the reference energy of the relaxed, bound crystal structure.


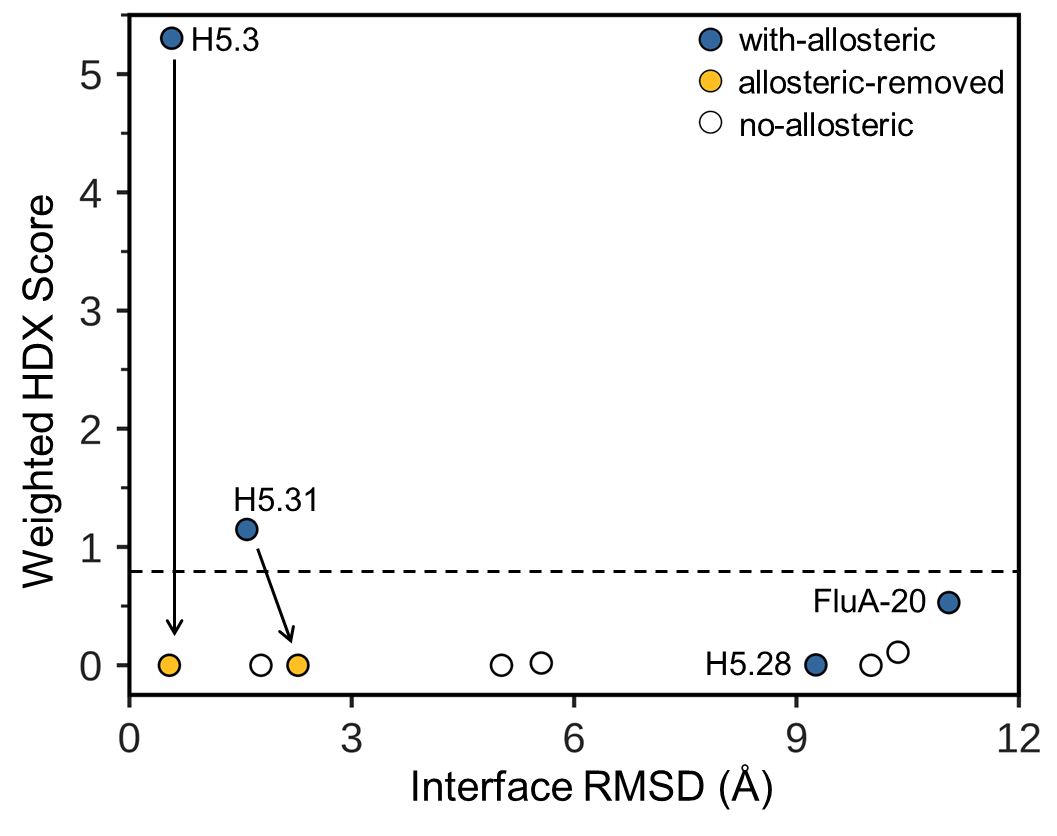


**Figure S12. Average weighted HDX score versus iRMSD of the top 10 scoring models generated by RosettaHDX starting with co-crystal structures.** The dashed line represents the prediction cutoff of 0.75, such that complexes with an average weighted HDX score above the line are predicted to contain allosteric peptides in their HDX dataset. Each point represents the top 10 scoring models of each benchmark complex. Complexes without allosteric peptides, with allosteric peptides, and with allosteric peptides identified and excluded are colored white, blue, and yellow, respectively. Arrows show the change in the selection of the top 10 scoring models for H5.3 and H5.31 (with corresponding changes in weighted HDX score and interface RMSD) when the allosteric peptide was identified and excluded from scoring.


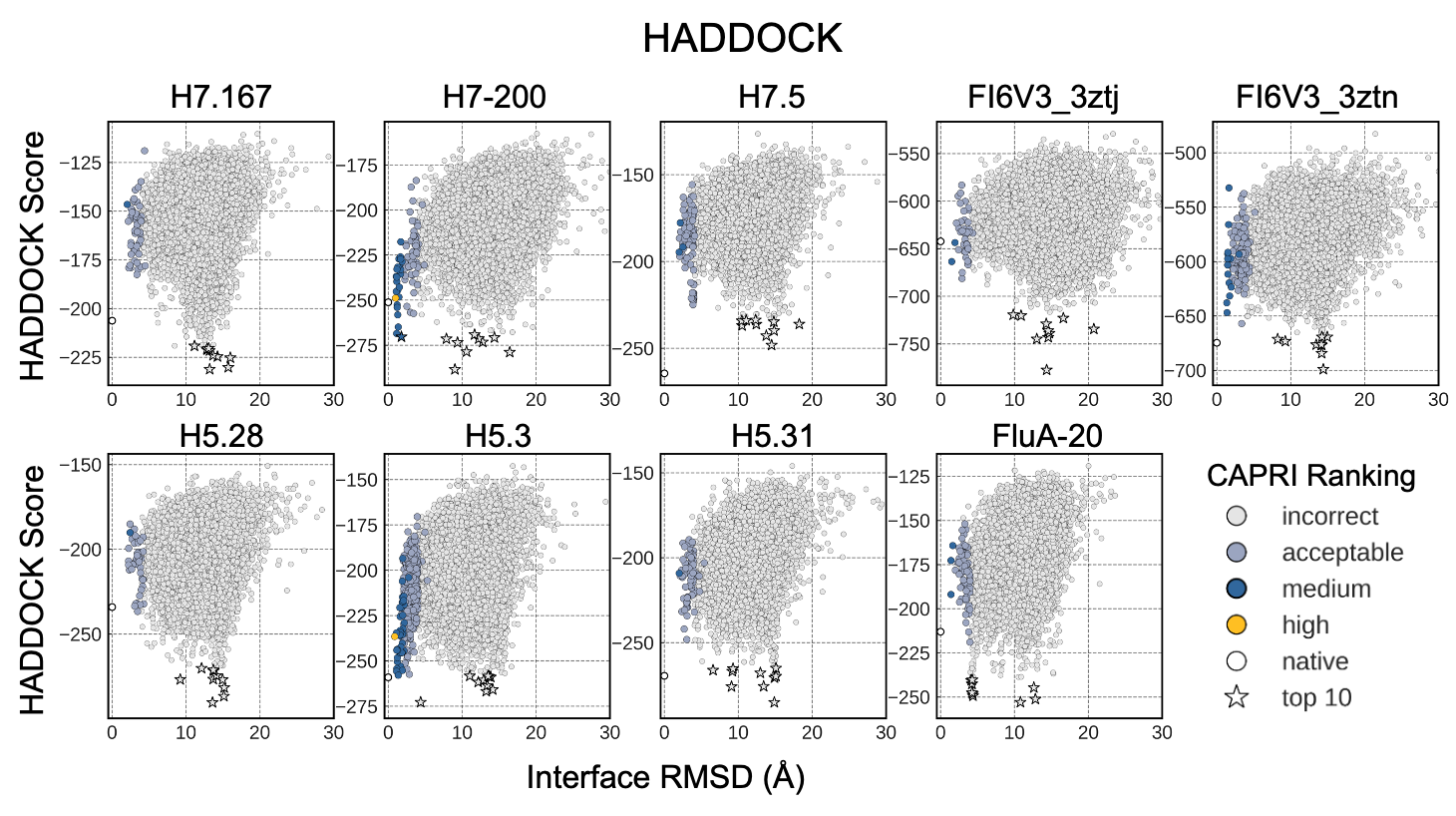


**Figure S13. HADDOCK score versus iRMSD for docking ensemble generated with HDX restraints starting from AF models.** The colors indicate model accuracy based on CAPRI criteria (Janin et al., 2003), as detailed in the legend: high (yellow), medium (dark blue), acceptable (light blue), and incorrect (light gray). For each benchmark complex, the top 10 scoring models selected by HADDOCK score are marked with star markers and colored according to the CAPRI criteria. The white circle represents the reference energy of the relaxed, bound crystal structure.


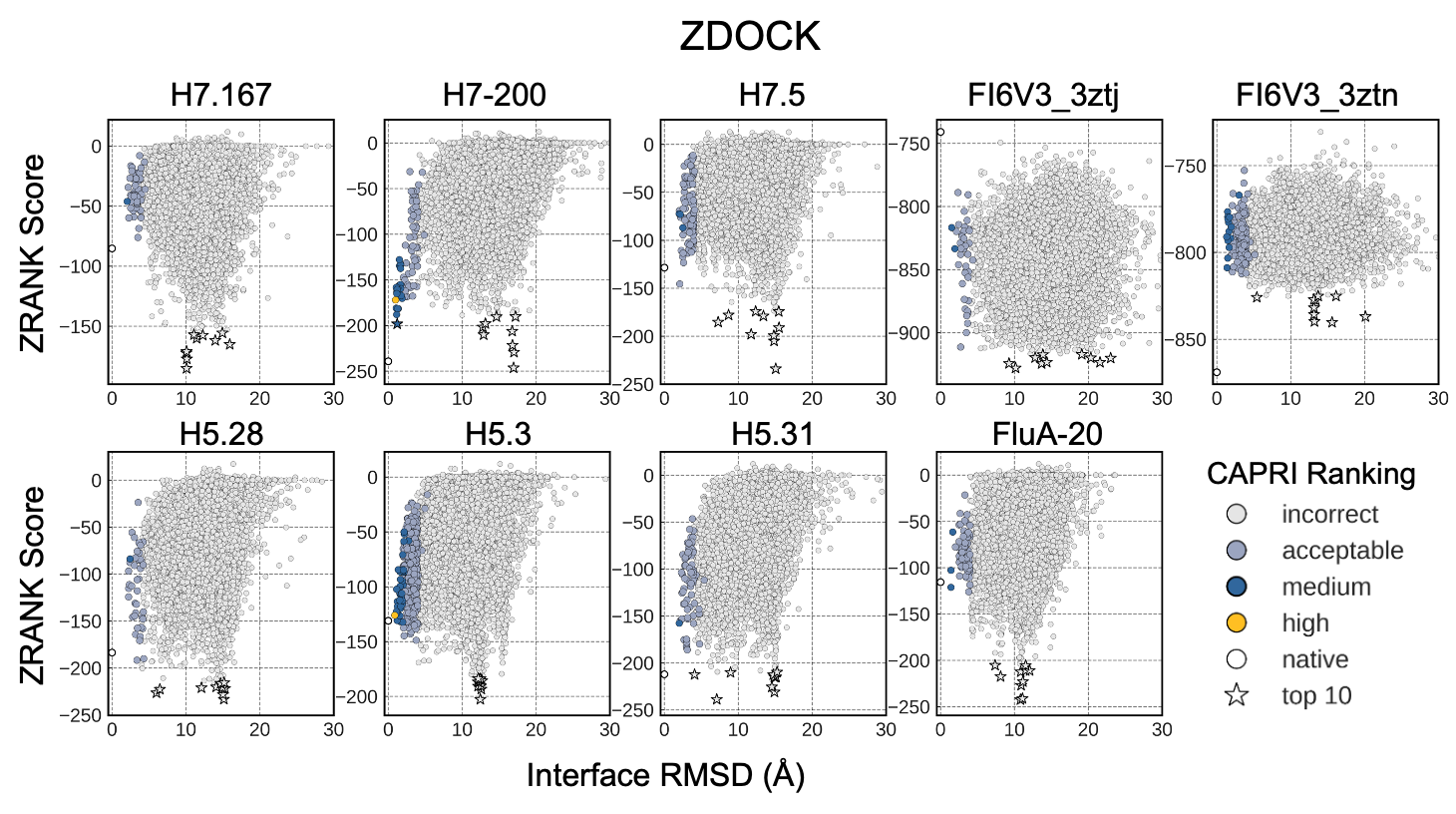


**Figure S14. ZRANK score (from ZDOCK) versus iRMSD for docking ensemble generated with HDX restraints starting from AF models.** The colors indicate model accuracy based on CAPRI criteria (Janin et al., 2003), as detailed in the legend: high (yellow), medium (dark blue), acceptable (light blue), and incorrect (light gray). For each benchmark complex, the top 10 scoring models selected by ZRANK score are marked with star markers and colored according to the CAPRI criteria. The white circle represents the reference energy of the relaxed, bound crystal structure.


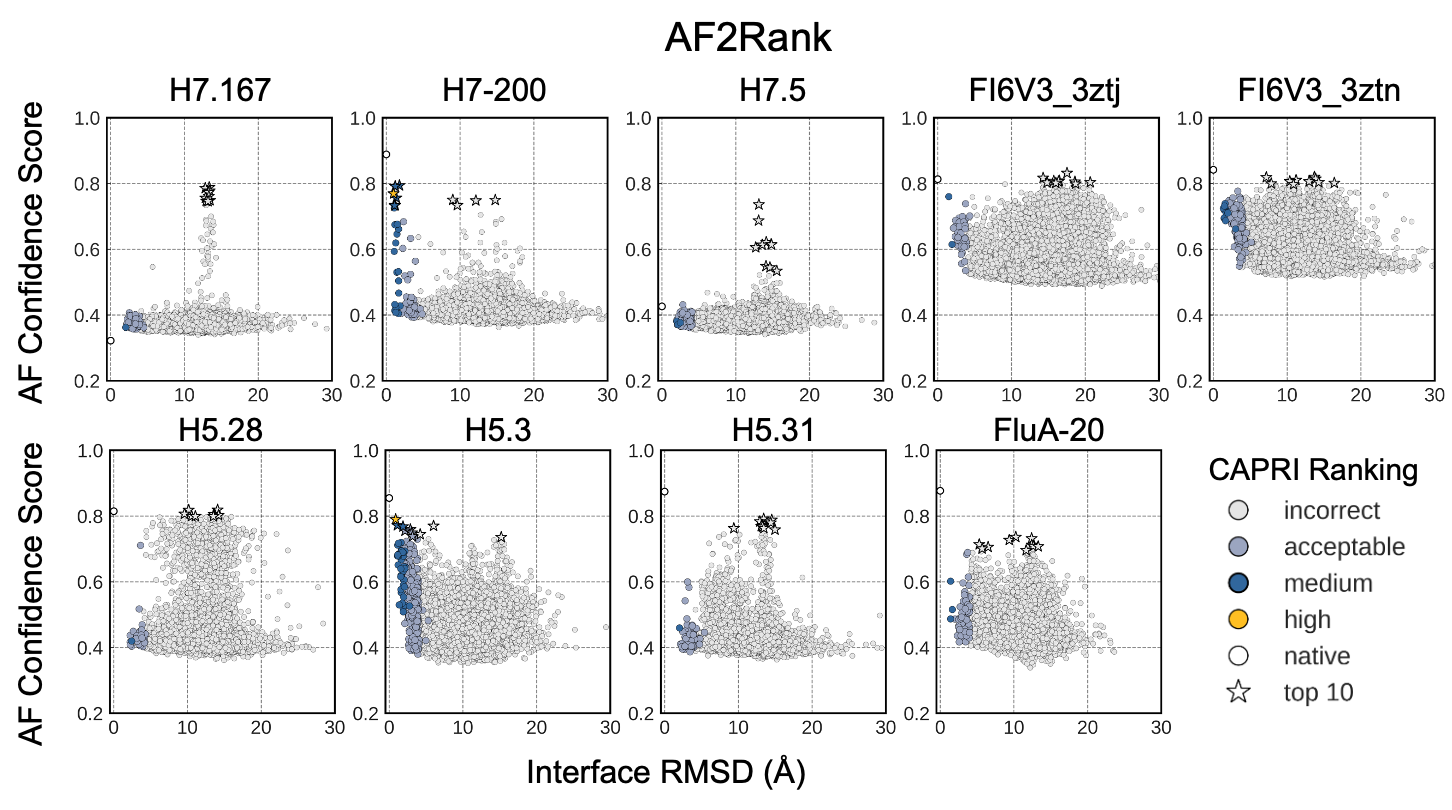


**Figure S15. AF confidence score (from AF2Rank) versus iRMSD for docking ensemble generated with HDX restraints starting from AF models.** The colors indicate model accuracy based on CAPRI criteria (Janin et al., 2003), as detailed in the legend: high (yellow), medium (dark blue), acceptable (light blue), and incorrect (light gray). For each benchmark complex, the top 10 scoring models selected by AF confidence score are marked with star markers and colored according to the CAPRI criteria. The white circle represents the reference energy of the relaxed, bound crystal structure.

**Additional Tables**

**Table S1. Summary of Ab-Ag benchmark complexes and corresponding HDX experimental results used in docking.** For each of the nine benchmark complexes, the PDB IDs, influenza hemagglutinin (HA) strains in experimentally determined structures, HA strains used in HDX experiments, and the potential interacting peptides identified from HDX experiments are reported. Allosteric peptides among potential interacting peptides as determined by HDX are marked with an asterisk *.

| **Ab** | **PDB ID and HA strain in co-crystal structure** | **HA strain in HDX data** | **HDX potential interacting peptide** |
| --- | --- | --- | --- |
| H7.167 | 5V2A  HA A/Shanghai/02/2013 | HA A/Shanghai/02/2013 (Zhang et al., 2013) | Peptide: 99-106 |
| H7-200 | 6UIG HA A/Shanghai/02/2013 | HA A/Shanghai/02/2013  (Dong et al., 2020) | Peptide: 154-172  [except 160P, 162P, 166P] |
| H7.5 | 6MLM HA A/New York/107/2003 | HA A/Anhui/DEWH72-01/2013 (unpublished work) | Peptide 1: 70-82 Peptide 2: 99-106 Peptide 3: 141-146 |
| FI6V3 | 3ZTJ HA A/Aichi/2/1968 | HA A/California/04/2009 (Garcia et al., 2023) | Peptide: 357-370 |
| FI6V3 | 3ZTN HA A/California/04/2009 | HA A/California/04/2009 (Garcia et al., 2023) | Peptide 1: 336-346  Peptide 2: 343-347  Peptide 3: 344-359  Peptide 4: 360-380 |
| H5.28 | 6P3S HA A/Vietnam/32/2004 | HA A/Vietnam/32/2004  (Bangaru et al., 2019) | Peptide 1: 41-50  [except 44P] Peptide 2: 84-95 * [except 88P] Peptide 3: 165-181 |
| H5.3 | 4XNQ HA A/Vietnam/32/2004 | HA A/Vietnam/32/2004  (unpublished work) | Peptide 1: 6-21 * [except 17P]  Peptide 2: 83-95  [except 88P]  Peptide 3: 137-142  Peptide 4: 168-180 |
| H5.31 | 6P3R HA A/Vietnam/32/2004 | HA A/Vietnam/32/2004  (unpublished work) | Peptide 1: 42-47  [except 44P]  Peptide 2: 83-95 *  [except 88P]  Peptide 3: 159-167  [except 163P]  Peptide 4: 169-180 |
| FluA-20 | 6OC3 HA A/Solomon Islands/3/2006 | HA A/Vietnam/32/2004 (unpublished work) | Peptide 1: 85-96 * [except 89P] Peptide 2: 160-168  [except 164P] Peptide 3: 169-181 |

**Table S2. Summary of docking results starting from co-crystal structures with and without HDX restraints for sampling and scoring**

|  |  | Without HDX restraints in sampling | | | | | HDX restraints in sampling | | | | |
| --- | --- | --- | --- | --- | --- | --- | --- | --- | --- | --- | --- |
|  |  | Top 1% iRMSD^a^ | without HDX score | | with HDX score | | Top 1%  iRMSD | without HDX score | | with HDX score | |
| Complex | No. of HDX Restraints |  | Top 10 Score^b^ | Enrich ment^c^ | Top 10 Score | Enrich ment |  | Top 10 Score | Enrich ment | Top 10 Score | Enrich ment |
| H7.167 | 1 | 4.0 | 12.6 | 1.6 | 9.7 | 3.3 | 3.4 | 10.2 | 1.3 | 10.0 | 1.7 |
| H7-200 | 1 | 3.5 | 4.0 | 2.2 | 3.5 | 2.8 | 3.1 | 5.0 | 2.0 | 5.0 | 2.2 |
| H7.5 | 3 | 4.0 | 4.8 | 2.1 | 2.5 | 3.5 | 2.3 | 1.8 | 2.3 | 1.8 | 2.9 |
| FI6V3_3ztj | 1 | 4.7 | 13.2 | 1.9 | 9.6 | 3.3 | 2.9 | 11.6 | 1.3 | 10.4 | 1.8 |
| FI6V3_3ztn | 4 | 4.5 | 8.6 | 1.5 | 6.9 | 3.0 | 2.1 | 7.5 | 2.4 | 5.6 | 2.6 |
| H5.28 | 3 | 4.9 | 11.0 | 2.5 | 7.9 | 3.8 | 3.4 | 9.3 | 1.7 | 9.3 | 1.7 |
| H5.3 | 4 | 3.9 | 12.0 | 1.6 | 7.9 | 3.4 | 0.7 | 0.5 | 2.8 | 0.6 | 3.5 |
| H5.31 | 4 | 4.6 | 7.3 | 2.1 | 3.1 | 4.3 | 2.3 | 2.3 | 1.7 | 1.6 | 2.6 |
| FluA-20 | 3 | 4.6 | 11.0 | 2.1 | 11.1 | 3.7 | 2.8 | 11.8 | 1.2 | 11.1 | 1.0 |
| Mean ^d^ | | 4.3 | 9.4 | 2.0 | 6.9 * | 3.5 ** | 2.6 ** | 6.7 | 1.8 | 6.2 * | 2.2 * |
| ≤ 1 Å ^e^ | |  | 15 | | 18 | |  | 34 | | 36 | |
| ≤ 2 Å ^e^ | |  | 24 | | 33 | |  | 37 | | 40 | |
| ≤ 4 Å ^e^ | |  | 30 | | 43 | |  | 42 | | 45 | |

^a^Top 1% iRMSD: average iRMSD of the best 1% of models ranked by iRMSD to the native crystal structure.

^b^Top 10 Score: average iRMSD of the top 10 scoring models, scored by either Rosetta interface score (binding energy) or the combined HDX score and Rosetta interface score.

^c^Enrichment: Enrichment value when models were scored using the standard Rosetta interface score alone versus with the addition of the HDX score.

^d^Mean: Average iRMSD and average enrichment for the entire benchmark set. The iRMSD of the top 1% of models generated with HDX restraints was compared to that of models generated without HDX restraints. Enrichment and average iRMSD of the top 10 models selected by the combined HDX score were compared to those selected by the Rosetta interface score. Wilcoxon two-tailed signed-rank test was employed (*p < 0.05, **p < 0.01). See also Figure 3 and S4 for statistical analysis.

^e^Number of models in the top 10 scoring models with iRMSD ≤ 1 Å (high quality), iRMSD ≤ 2 Å (medium quality or better), and iRMSD ≤ 4 Å (acceptable quality or better) for the entire benchmark set.

**Table S3. Number of near-native models (CAPRI high, medium, and acceptable quality) generated with and without HDX restraints for each of the nine benchmark complexes.** The counts are shown for docking simulations using crystal structures as input and for docking simulations using AF Ab and Ag models as input.

| Crystal structures as docking input | Without HDX restraints | | | With HDX restraints | | |
| --- | --- | --- | --- | --- | --- | --- |
| Complex | High | Medium | Acceptable | High | Medium | Acceptable |
| H7.167 | 0 | 2 | 41 | 0 | 4 | 71 |
| H7-200 | 9 | 11 | 34 | 11 | 10 | 48 |
| H7.5 | 8 | 5 | 21 | 18 | 21 | 75 |
| FI6V3_3ztj | 1 | 12 | 14 | 2 | 28 | 49 |
| FI6V3_3ztn | 3 | 1 | 26 | 13 | 13 | 193 |
| H5.28 | 0 | 6 | 10 | 1 | 7 | 64 |
| H5.3 | 7 | 12 | 24 | 93 | 112 | 222 |
| H5.31 | 2 | 9 | 14 | 14 | 31 | 69 |
| FluA-20 | 0 | 1 | 23 | 5 | 13 | 123 |
| Total | 30 | 59 | 207 | 157 * | 239 * | 914 ** |
| AF models as docking input | Without HDX restraints | | | With HDX restraints | | |
| Complex | High | Medium | Acceptable | High | Medium | Acceptable |
| H7.167 | 0 | 0 | 18 | 0 | 1 | 51 |
| H7-200 | 0 | 13 | 45 | 1 | 23 | 59 |
| H7.5 | 0 | 3 | 30 | 0 | 3 | 91 |
| FI6V3_3ztj | 0 | 3 | 10 | 0 | 2 | 38 |
| FI6V3_3ztn | 0 | 3 | 18 | 0 | 14 | 125 |
| H5.28 | 0 | 1 | 19 | 0 | 1 | 40 |
| H5.3 | 0 | 11 | 39 | 1 | 41 | 289 |
| H5.31 | 0 | 1 | 18 | 0 | 1 | 70 |
| FluA-20 | 0 | 2 | 19 | 0 | 3 | 65 |
| Total | 0 | 37 | 216 | 2 | 89 | 828 * |

**Table S4. Summary of docking results starting from AF models with and without HDX restraints for sampling and scoring**

|  |  | No HDX restraints in sampling | | | | | HDX restraints in sampling | | | | |
| --- | --- | --- | --- | --- | --- | --- | --- | --- | --- | --- | --- |
|  |  | Top 1%  iRMSD^a^ | without HDX score | | with HDX score | | Top 1%  iRMSD | without HDX score | | with HDX score | |
| Complex | No. of HDX Restraints |  | Top 10 Score^b^ | Enrich ment^c^ | Top 10 Score | Enrich ment |  | Top 10 Score | Enrich ment | Top 10 Score | Enrich ment |
| H7.167 | 1 | 4.6 | 12.6 | 1.2 | 10.6 | 2.5 | 3.8 | 12.0 | 1.1 | 10.8 | 1.5 |
| H7-200 | 1 | 3.6 | 13.8 | 1.8 | 12.6 | 2.5 | 3.0 | 9.1 | 1.9 | 7.8 | 2.2 |
| H7.5 | 3 | 4.4 | 11.0 | 1.8 | 7.4 | 3.4 | 3.4 | 7.1 | 1.7 | 5.6 | 2.7 |
| FI6V3_3ztj | 1 | 5.6 | 22.9 | 1.6 | 14.7 | 3.0 | 4.1 | 14.2 | 0.9 | 15.0 | 1.6 |
| FI6V3_3ztn | 4 | 4.9 | 19.9 | 1.6 | 14.4 | 3.2 | 2.9 | 12.6 | 0.8 | 13.1 | 0.9 |
| H5.28 | 3 | 5.1 | 11.8 | 2.0 | 9.8 | 3.5 | 4.0 | 11.7 | 1.8 | 9.6 | 1.9 |
| H5.3 | 4 | 3.8 | 8.4 | 1.6 | 5.0 | 3.4 | 2.0 | 9.5 | 1.4 | 10.7 | 1.8 |
| H5.31 | 4 | 5.0 | 14.1 | 2.2 | 11.2 | 4.1 | 3.6 | 12.1 | 1.6 | 10.8 | 2.1 |
| FluA-20 | 3 | 4.6 | 12.1 | 1.6 | 10.1 | 3.3 | 3.5 | 10.2 | 1.2 | 9.4 | 1.2 |
| Mean ^d^ | | 4.6 | 14.1 | 1.7 | 10.6 ** | 3.4 ** | 2.6 ** | 10.9 | 1.4 | 10.3 * | 1.8 * |
| ≤ 1 Å ^e^ | |  | 0 | | 0 | |  | 0 | | 0 | |
| ≤ 2 Å ^e^ | |  | 3 | | 5 | |  | 6 | | 5 | |
| ≤ 4 Å ^e^ | |  | 0 | | 7 | |  | 11 | | 12 | |

^a^Top 1% iRMSD: average iRMSD of the best 1% of models ranked by iRMSD to the native crystal structure.

^b^Top 10 Score: average iRMSD of the top 10 scoring models, scored by either Rosetta interface score (binding energy) or the combined HDX score and Rosetta interface score.

^c^Enrichment: Enrichment value when models were scored using the standard Rosetta interface score alone versus with the addition of the HDX score.

^d^Mean: Average iRMSD and average enrichment for the entire benchmark set. The iRMSD of the top 1% of models generated with HDX restraints was compared to that of models generated without HDX restraints. Enrichment and average iRMSD of the top 10 models selected by the combined HDX score were compared to those selected by the Rosetta interface score. Wilcoxon two-tailed signed-rank test was employed (*p < 0.05, **p < 0.01). See also Figure S8 and S10 for statistical analysis.

^e^Number of models in the top 10 scoring models with iRMSD ≤ 1 Å (high quality), iRMSD ≤ 2 Å (medium quality or better), and iRMSD ≤ 4 Å (acceptable quality or better) for the entire benchmark set.

**Table S5. Average weighted HDX score of the top 10 scoring model generated with RosettaHDX.** The allosteric peptide ID and the original average weighted HDX score (considering all HDX peptides) are reported for each of the nine benchmark complexes, in RosettaHDX docking simulations starting from crystal structures and in RosettaHDX docking simulations starting from AF models. For complexes with an average weighted HDX score > 0.75 (such as H5.3 and H5.31), each HDX peptide from the HDX set was sequentially excluded from HDX score; the new top 10 models were selected; and the new average weighted HDX scores after each peptide exclusion was reported.

| Crystal structures as docking input | | Weighted HDX score of top 10 scoring | | | | |
| --- | --- | --- | --- | --- | --- | --- |
| Complex | Allosteric peptide | All HDX peptides | Exclude pep 1 | Exclude pep 2 | Exclude pep 3 | Exclude pep 4 |
| H7.167 | N/A | 0 |  |  |  |  |
| H7-200 | N/A | 0 |  |  |  |  |
| H7.5 | N/A | 0 |  |  |  |  |
| FI6V3_3ztj | N/A | 0.11 |  |  |  |  |
| FI6V3_3ztn | N/A | 0.02 |  |  |  |  |
| H5.28 | Peptide 2 | 0 |  |  |  |  |
| H5.3 | Peptide 1 | 5.3 | 0 | 5.87 | 6.75 | 6.23 |
| H5.31 | Peptide 2 | 1.15 | 1.47 | 0 | 1.69 | 1.28 |
| FluA-20 | Peptide 1 | 0.53 |  |  |  |  |
| AF models as docking input | | Weighted HDX score of top 10 scoring | | | | |
| Complex | Allosteric peptide | All HDX peptides | Exclude pep 1 | Exclude pep 2 | Exclude pep 3 | Exclude pep 4 |
| H7.167 | N/A | 0.38 |  |  |  |  |
| H7-200 | N/A | 0.09 |  |  |  |  |
| H7.5 | N/A | 0 |  |  |  |  |
| FI6V3_3ztj | N/A | 0.72 |  |  |  |  |
| FI6V3_3ztn | N/A | 0.04 |  |  |  |  |
| H5.28 | Peptide 2 | 0.06 |  |  |  |  |
| H5.3 | Peptide 1 | 3.94 | 0 | 2.37 | 0.63 | 2.79 |
| H5.31 | Peptide 2 | 1.67 | 0.93 | 0.58 | 0.71 | 1.23 |
| FluA-20 | Peptide 1 | 0.06 |  |  |  |  |

**Protocol Capture**

**RosettaHDX tutorial**

The RosettaHDX protocol is designed to generate docking models of antibody-antigen (Ab-Ag) complex and select them using guidance from experimental HDX-MS data, enhancing the quality of both the generated docking ensemble and the selected top-scoring models. RosettaHDX is used when the Ab-Ag complex of interest has been characterized using HDX-MS but lacks a co-crystal structure.

If unbound Ab and Ag structures are available from crystallization, they can be used; otherwise, they can be modeled using AlphaFold2 (AF2) as we did in this manuscript. AF2 can be installed following the instructions provided at https://github.com/deepmind/alphafold. After installation, unbound Ab and Ag are modeled by providing their protein sequences in FASTA format. For the Ab which contains both heavy and light chains, the --model_preset option is set to "multimer", since the default option is "monomer". Aside from this adjustment, all default settings can be used for AF2 runs. It's important to note that AF2 is just one approach for obtaining unbound Ab and Ag structures. Other options, such as *de novo* modeling or homology modeling can also be employed. Specifically, for Ab modeling, several deep learning-based methods are available, such as DeepAb, IgFold, and OmegaFold (Ruffolo and Gray, 2022; Ruffolo et al., 2022; Wu et al., 2022).

In this tutorial, user-defined variables are indicated within angle brackets (< >). To perform the RosettaHDX protocol to dock antibody (Ab) and antigen (Ag) with HDX-MS experimental data, five main stages need to be performed in Rosetta:

1. Generate the HDX restraint file in Rosetta

2. Refine structures/models of the unbound Ab and Ag

3. Generate a docking ensemble with HDX

4. Rescore the docked models with HDX

5. Predict the allosteric peptide

**Part 1: Generate the HDX restraint file in Rosetta**

A complete HDX restraint file contains one AmbiguousConstraint for each HDX-interacting peptide identified on the Ag. Each AmbiguousConstraint applies a flat harmonic Rosetta restraint from one HDX peptide to the Ab complementarity-determining regions (CDRs). Below is a snippet example of an AmbiguousConstraint between HDX peptide 42-47 and the Ab CDRs (HCDR1: 240-244, HCDR2: 259-275, HCDR3: 308-327, LCDR1: 361-371, LCDR2: 387-393, LCDR3: 426-436) for demonstration.

AmbiguousConstraint

AtomPair CA 42 CA 240 FLAT_HARMONIC 0 1 10

AtomPair CA 42 CA 241 FLAT_HARMONIC 0 1 10

AtomPair CA 42 CA 243 FLAT_HARMONIC 0 1 10 Restrained to HCDR1

AtomPair CA 42 CA 244 FLAT_HARMONIC 0 1 10

AtomPair CA 42 CA 244 FLAT_HARMONIC 0 1 10

AtomPair CA 42 CA 259 FLAT_HARMONIC 0 1 10

... Restrained to HCDR2

AtomPair CA 42 CA 275 FLAT_HARMONIC 0 1 10

AtomPair CA 42 CA 308 FLAT_HARMONIC 0 1 10

... Restrained to HCDR3

AtomPair CA 42 CA 327 FLAT_HARMONIC 0 1 10

AtomPair CA 42 CA 361 FLAT_HARMONIC 0 1 10

... Restrained to LCDR1

AtomPair CA 42 CA 371 FLAT_HARMONIC 0 1 10

AtomPair CA 42 CA 387 FLAT_HARMONIC 0 1 10

... Restrained to LCDR2

AtomPair CA 42 CA 393 FLAT_HARMONIC 0 1 10

AtomPair CA 42 CA 426 FLAT_HARMONIC 0 1 10

... Restrained to LCDR3

AtomPair CA 42 CA 436 FLAT_HARMONIC 0 1 10

# Do the same for remaining residues in HDX peptide 42-47

END

A complete restraint file (HDX_restraint.cst) is provided in the Supplementary File for reference.

**Part 2: Refine structures/models of the unbound Ab and Ag**

1. If the unbound Ab and Ag are crystal structures, remove metal ions and other ligands from the pdb files using the clean_pdb.py script in Rosetta, by running the following command.

/path/to/rosetta/protein_tools/scripts/clean_pdb.py <pdb_file> <chains>

- <pdb_file> pdb file of the unbound Ab or Ag
- <chains> chains of the Ab or Ag. Ex: A for the Ag chain, HL are the Ab heavy and light chains

2. Create a text file listing the paths to each unbound Ab and Ag structure/model to be refined. For example:

/path/to/file/Ab_ranked_0.pdb

/path/to/file/Ab_ranked_1.pdb

/path/to/file/Ab_ranked_2.pdb

/path/to/file/Ag_ranked_3.pdb

/path/to/file/Ag_ranked_4.pdb

/path/to/file/Ab_ranked_0.pdb

/path/to/file/Ab_ranked_1.pdb

/path/to/file/Ab_ranked_2.pdb

/path/to/file/Ab_ranked_3.pdb

/path/to/file/Ab_ranked_4.pdb

3. Refine the unbound Ab and Ag structures/models using FastRelax by running the following command.

/path/to/rosetta/main/source/bin/relax.default.linuxgccrelease -in:file:l <unbound_list.txt> -use_input_sc -nstruct 1 -relax:fast -relax:constrain_relax_to_start_coords

- <unbound_list.txt> the text file listing the unbound Abs and Ags created in step 2

4. Prepare pdb files of predock starting conformations, each contains one relaxed Ab and one relaxed Ag model assembled in PyMOL

5. Prepack the predock starting conformations to resolve any clashes at the interface by running the following command.

/path/to/rosetta/main/source/bin/docking_prepack_protocol.default.linuxgccrelease -in:file:l <predock_list.txt> -nstruct 1 -partners <chains>

- <predock_list.txt> the text file listing the starting conformation pdbs created in step 4
- <chains> chains of the Ab and Ag. Ex: A_HL where A is the Ag chain, H and L are the Ab heavy and light chains

**Part 3: Generate a docking ensemble with HDX by running the following command.**

/path/to/rosetta/main/source/bin/rosetta_scripts.linuxgccrelease -database /path/to/rosetta/main/database/ -in:file:l <prepacked_list.txt> -nstruct <n_structs> -parser:protocol HDX_docking.xml @docking.options -out:file:scorefile docking.fasc

- <prepacked_list.txt> the text file listing the prepacked starting conformation pdbs created in step 5 of Part 2
- <n_structs> the number of models to be generated for each starting conformation (a total of ≥10,000 models is recommended)

An input Rosetta XML script (HDX_docking.xml) as well as an options file (docking.options)are provided in the Supplementary File.

**Part 4: Rescore the docked models with HDX**

1. Compute HDX scores for the docked models by running the following command

/path/to/rosetta/main/source/bin/score.linuxgccrelease -in:file:l <docked_model_list.txt> -constraints:cst_file <HDX_restraint_file> -score:set_weights atom_pair_constraint 1.0 -score:weights none -out:file:scorefile HDX_sc.fasc

- <docked_model_list.txt> the text file listing the docking models generated in Part 3
- <HDX_restraint_file> the HDX restraint file generated in step 1 of Part 1

2. The Rosetta interface score (dG_separated) and the raw HDX score can be extracted from the score file by running the following commands

cat docking.fasc | tail -n+2 | awk '{print $NF, $13}' > docking.csv

cat HDX_sc.fasc | awk '{print $NF, $3}' > HDX_sc.csv

3. Finally, the combined Rosetta interface and HDX score and the average weighted HDX score can be extracted from the two score files above by using the python script calc_HDX_wt.py provided in the Supplementary File

python calc_HDX_wt.py --docking_csv docking.csv --HDX_sc_csv HDX_sc.csv

Step 3 will result in an output file combined_Rosetta_HDX_sc.csv containing the Rosetta_interface_sc, weighted_HDX_sc, and combined_Rosetta_HDX_sc for each of the docked models. Use the combined_Rosetta_HDX_sc value to sort the poses and thus select the top scoring models

description Rosetta_interface_sc weighted_HDX_sc combined_Rosetta_HDX_sc

model_1 -13.875 0.739 -13.134

model_2 -8.383 66.065 57.682

…

model_10000 -43.203 0.509 -42.694

The average weighted HDX score of the top 10 scoring models will also be printed out (e.g., “Average weighted HDX score of top 10 scoring: 1.67”). If this value exceeds the 0.75 threshold, proceed to Stage 5 to predict the allosteric peptide

**Part 5: Predict allosteric peptides**

1. Generate HDX restraint files for each HDX peptide using the same format as in Part 1. Name these files as HDX_pep1.cst, HDX_pep2.cst, HDX_pep3.cst, and so on. Example individual HDX constraint files are provided in the Supplementary File

2. Compute penalty scores derived from each HDX peptide for the docked models by running the following commands

/path/to/rosetta/main/source/bin/score.linuxgccrelease -in:file:l <docked_model_list.txt> -constraints:cst_file <HDX_pepX_restraint> -score:set_weights atom_pair_constraint 1.0 -score:weights none -out:file:scorefile <HDX_pepX.sc>

- <docked_model_list.txt> the text file listing the docking models generated in Part 3
- <HDX_pepX_restraint> HDX restraint file of each peptide generated in step 1

· <HDX_pepX.sc> the penalty score file for each HDX peptide

ls HDX_pep*.fasc | awk '{print "cat "$1" | awk '\''{print $NF, $3}'\'' > "substr($1, 0, length($1)-5)".csv"}' > fasc_to_csv.sh

sh fasc_to_csv.sh

3. To identify the specific allosteric peptide within the HDX dataset, each peptide is sequentially excluded from the HDX score. This updated HDX score was combined with the Rosetta interface score to select a new set of top 10 scoring models. The average weighted HDX score for this updated set was evaluated. This can be done using the python script allosteric_peptide.py provided in the Supplementary File

python allosteric_peptide.py --docking_csv docking.csv --HDX_sc_csv HDX_sc.csv --HDX_pep_list <HDX_pep.txt>

- <HDX_pep.txt> the text file listing score csv files of each HDX peptide generated in step 2

Step 3 will print out the updated average weighted HDX score for each peptide exclusion. For example:

Average weighted HDX score of top 10 scoring when excluding pep1: 1.47

Average weighted HDX score of top 10 scoring when excluding pep2: 0.58

Average weighted HDX score of top 10 scoring when excluding pep3: 1.69

Average weighted HDX score of top 10 scoring when excluding pep4: 1.28

For the peptide exclusion that yields the lowest average weighted HDX score, a new_combined_Rosetta_HDX_sc.csv file will be generated to reflect the updated combined_Rosetta_HDX_sc values for re-selecting the top scoring models.

If the updated average HDX score remains above 0.75 after the first peptide exclusion, this indicates multiple allosteric peptides. In such cases, update the HDX restraints in Part 1 after each allosteric peptide identification, then proceed with redocking and rescoring.

# References

Bangaru, S., Lang, S., Schotsaert, M., Vanderven, H.A., Zhu, X., Kose, N., Bombardi, R., Finn, J.A., Kent, S.J., Gilchuk, P., Gilchuk, I., Turner, H.L., García-Sastre, A., Li, S., Ward, A.B., Wilson, I.A., Crowe, J.E., Jr., 2019. A Site of Vulnerability on the Influenza Virus Hemagglutinin Head Domain Trimer Interface. Cell 177, 1136-1152.e1118.

Dong, J., Gilchuk, I., Li, S., Irving, R., Goff, M.T., Turner, H.L., Ward, A.B., Carnahan, R.H., Crowe, J.E., Jr., 2020. Anti-influenza H7 human antibody targets antigenic site in hemagglutinin head domain interface. J Clin Invest 130, 4734-4739.

Garcia, N.K., Kephart, S.M., Benhaim, M.A., Matsui, T., Mileant, A., Guttman, M., Lee, K.K., 2023. Structural dynamics reveal subtype-specific activation and inhibition of influenza virus hemagglutinin. J Biol Chem 299, 104765.

Janin, J., Henrick, K., Moult, J., Eyck, L.T., Sternberg, M.J.E., Vajda, S., Vakser, I., Wodak, S.J., 2003. CAPRI: A Critical Assessment of PRedicted Interactions. Proteins: Structure, Function, and Bioinformatics 52, 2-9.

Ruffolo, J.A., Gray, J.J., 2022. Fast, accurate antibody structure prediction from deep learning on massive set of natural antibodies. Biophysical Journal 121, 155a-156a.

Ruffolo, J.A., Sulam, J., Gray, J.J., 2022. Antibody structure prediction using interpretable deep learning. Patterns 3, 100406.

Wu, R., Ding, F., Wang, R., Shen, R., Zhang, X., Luo, S., Su, C., Wu, Z., Xie, Q., Berger, B., Ma, J., Peng, J., 2022. High-resolution de novo structure prediction from primary sequence. bioRxiv, 2022.2007.2021.500999.

Zhang, Q., Shi, J., Deng, G., Guo, J., Zeng, X., He, X., Kong, H., Gu, C., Li, X., Liu, J., Wang, G., Chen, Y., Liu, L., Liang, L., Li, Y., Fan, J., Wang, J., Li, W., Guan, L., Li, Q., Yang, H., Chen, P., Jiang, L., Guan, Y., Xin, X., Jiang, Y., Tian, G., Wang, X., Qiao, C., Li, C., Bu, Z., Chen, H., 2013. H7N9 influenza viruses are transmissible in ferrets by respiratory droplet. Science 341, 410-414.
